# Class II phosphoinositide 3-kinase C2β regulates a novel signaling pathway involved in breast cancer progression

**DOI:** 10.18632/oncotarget.7761

**Published:** 2016-02-26

**Authors:** Anissa Chikh, Riccardo Ferro, Jonathan J. Abbott, Roberto Piñeiro, Richard Buus, Manuela Iezzi, Francesca Ricci, Daniele Bergamaschi, Paola Ostano, Giovanna Chiorino, Rossano Lattanzio, Massimo Broggini, Mauro Piantelli, Tania Maffucci, Marco Falasca

**Affiliations:** ^1^ Queen Mary University of London, Barts and The London School of Medicine and Dentistry, Blizard Institute, Centre for Cell Biology and Cutaneous Research, London, UK; ^2^ Department of Medical, Oral and Biotechnological Sciences, University “G. d'Annunzio”, Chieti, Italy; ^3^ Aging Research Centre (Ce.S.I.), Foundation University “G. d'Annunzio”, Chieti, Italy; ^4^ Laboratory of Molecular Pharmacology IRCCS-Istituto di Ricerche Farmacologiche “Mario Negri”, Milan, Italy; ^5^ Cancer Genomics Laboratory, Fondazione Edo and Elvo Tempia, Biella, Italy; ^6^ Metabolic Signalling Group, School of Biomedical Sciences, CHIRI Biosciences, Curtin University, Perth, Western Australia, Australia

**Keywords:** breast cancer, cyclinB1, metastasis, miR449, phosphoinositide 3-kinase C2β

## Abstract

It is now well established that the enzymes phosphoinositide 3-kinases (PI3Ks) have a key role in the development and progression of many cancer types and indeed PI3Ks inhibitors are currently being tested in clinical trials. Although eight distinct PI3K isoforms exist, grouped into three classes, most of the evidence currently available are focused on one specific isoform with very little known about the potential role of the other members of this family in cancer. Here we demonstrate that the class II enzyme PI3K-C2β is overexpressed in several human breast cancer cell lines and in human breast cancer specimens. Our data indicate that PI3K-C2β regulates breast cancer cell growth *in vitro* and *in vivo* and that PI3K-C2β expression in breast tissues is correlated with the proliferative status of the tumor. Specifically we show that downregulation of PI3K-C2β in breast cancer cell lines reduces colony formation, induces cell cycle arrest and inhibits tumor growth, in particular in an estrogen-dependent *in vivo* xenograft. Investigation of the mechanism of the PI3K-C2β-dependent regulation of cell cycle progression and cell growth revealed that PI3K-C2β regulates cyclin B1 protein levels through modulation of microRNA miR-449a levels. Our data further demonstrate that downregulation of PI3K-C2β inhibits breast cancer cell invasion *in vitro* and breast cancer metastasis *in vivo*. Consistent with this, PI3K-C2β is highly expressed in lymph-nodes metastases compared to matching primary tumors. These data demonstrate that PI3K-C2β plays a pivotal role in breast cancer progression and in metastasis development. Our data indicate that PI3K-C2β may represent a key molecular switch that regulates a rate-limiting step in breast tumor progression and therefore it may be targeted to limit breast cancer spread.

## INTRODUCTION

Regulation of cell proliferation and cell survival in breast cancer is a complex interplay between steroid hormones, growth factors and their receptors [1]. Understanding the signaling pathways involved in these processes may help find predictive factors for tumor aggressiveness and therapy resistance. Among the different pathways activated by growth factors receptors, signals transmitted by the phosphoinositide 3-kinase (PI3K)/Akt pathway have proven important for cell survival in many cell types [2]. Several lines of evidence indicate that an inappropriate activation of the PI3K/Akt pathway is linked to the development of cancer and the inhibition of this pathway has been shown to facilitate apoptosis and to sensitize cells to cytotoxic drugs in experimental studies [3, 4]. It is therefore not surprising that the PI3K/Akt pathway is currently an attractive target for therapeutic strategies. PI3K is a family of enzymes, comprising eight mammalian isoforms grouped into three classes [5]. The majority of studies on PI3K involvement in cancer have been focused so far on members of the class I subfamily. A broad range of tumor types, including breast cancer, are characterized by activating mutations of *PIK3CA* (the gene encoding for p110α, a member of the class I group) and its downstream effector AKT1, as well as inactivating mutations of phosphatase and tensin homolog (*PTEN*), the gene encoding the lipid phosphatase that counteracts the action of PI3Ks [6]. Class I PI3Ks, in particular p110α, have therefore a well-established role in cancer development and progression. In contrast, the potential role of the class II PI3Ks in cancer has not been fully explored, although accumulating evidence now suggests that this class may also play a role in cancer development [7]. For instance, gene expression profiling has revealed an increased expression of the β isoform of the class II group (PI3K-C2β) in several cancers such as acute myeloid leukemia, glioblastoma and acute lymphocytic leukemia [8-10]. Consistent with this, PI3K-C2β was found to be overexpressed in subsets of tumor and cell lines from acute myeloid leukemia, glioblastoma multiforme, medulloblastoma and small cell lung cancer [11]. It has also been shown that different small cell lung cancer cell lines overexpress distinct subsets of class IA and II PI3Ks, which results in differences in the signaling cascades activated by stem cell factor [12]. Increased levels of PI3K-C2β have also been detected in primary neuroblastoma tumors and cell lines [11, 13]. Recently, a significant association has been observed between a cluster of variants located upstream and in the promoter regions of *PIK3C2B*, the gene encoding for PI3K-C2β, and prostate cancer risk, especially for familial, early-onset disease [14]. In addition, significant recurrent mutations (12.9%) have also been found in *PIK3C2B* in lung cancer [15]. Other evidence supporting a role for PI3K-C2β in cancer includes our demonstration that activation of this enzyme is necessary for lysophosphatidic-dependent migration of ovarian and cervical cancer cells [16]. Similarly, it was reported that overexpression of PI3K-C2β enhances migration of A-431 epidermoid carcinoma cells, while overexpression of dominant negative PI3K-C2β reduces this process [17]. More recently, it has been shown that PI3K-C2β has a key role in neuroblastoma tumorigenesis [18]. Taken together, these data suggested that PI3K-C2β may play a role in cancer development. Interestingly, data also indicated that this isoform may be involved in epidermal growth factor signaling [19], but the precise physiological role of PI3K-C2β in this context and the potential correlation to cancer development have not been investigated.

In this study, we demonstrate that PI3K-C2β is overexpressed in several human breast cancer cell lines and in human breast cancer specimens. Our data indicate that PI3K-C2β regulates breast cancer cell growth and that PI3K-C2β expression in breast tissues is correlated with the proliferative status of the tumor. Furthermore, downregulation of PI3K-C2β inhibits breast cancer cell invasion *in vitro* and breast cancer metastasis formation *in vivo*. This establishes PI3K-C2β as a promising target in breast cancer progression and in metastasis development.

## RESULTS AND DISCUSSION

### PI3K-C2β is overexpressed in human breast cancer cell lines and regulates breast cancer cell tumorigenesis

**Figure 1:**
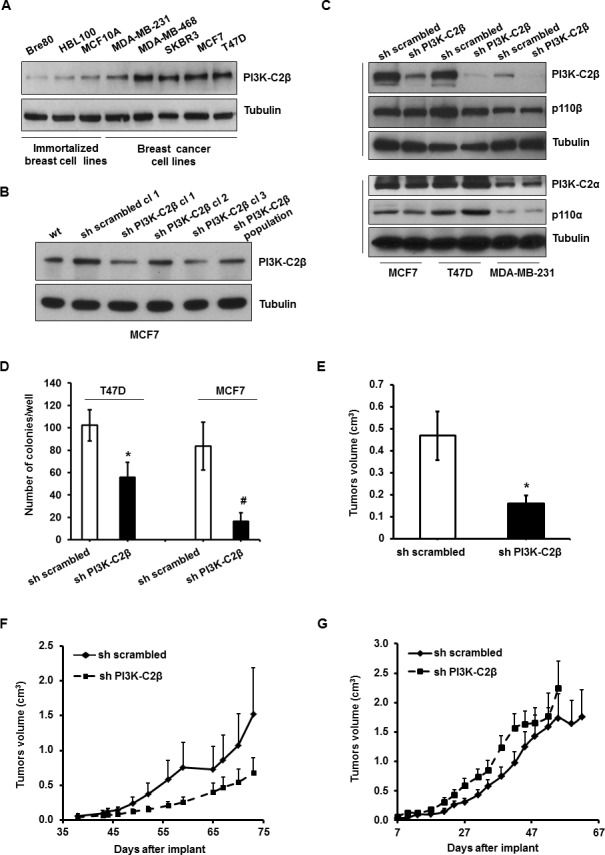
PI3K-C2β is overexpressed in human breast cancer cell lines and plays a critical role in breast cancer cell tumorigenesis **A.**Western blot analysis of PI3K-C2β expression in immortalized breast cell lines and in breast cancer cell lines. Tubulin was used as a loading control. **B.** Expression levels of PI3K-C2β in parental MCF7 (wt) and in the indicated MCF-7 cell lines. Tubulin was used as loading control. **C.** Levels of p110α, p110β, PI3K-C2α and PI3K-C2β in MCF7, T47D and MDA-MB-231 stable cell lines. In each set of gels, tubulin was used as loading control. **D.** Results from soft agar colony formation assays performed in the indicated T47D and MCF7 cell lines. Data indicate the number of colonies/well and are means ± s.e.m. of *n* = 3 independent experiments performed in triplicate. *p = 0.025, #*p* = 0.030 (*t*-Test, one tailed distribution, paired). **E.** The indicated MCF7 cells were diluted in medium+Matrigel™ and injected into the mammary fat pad of 5 week-old pathogen-free nude mice on day 0. E_2_ pellets were injected subcutaneously into the neck with pellet trochar. Tumors volume was measured after 51 days of implantation. Data are means ± s.e.m. from *n* = 6 (sh scrambled) and *n* = 16 (sh PI3K-C2β) mice. *p = 0.019 (*t*-Test, one tailed distribution, two sample unequal variance). **F.** Control MDA-MB-231 (sh scrambled) and MDA-MB-231 lacking PI3K-C2β (sh PI3K-C2β) were injected in the mammary fat pad in female immunodeficient mice. Tumor weight was assessed at the indicated days after injection. Values are means ± SD from 9 mice per group. **G.** sh scrambled and sh PI3K-C2β MDA-MB-231 cells were injected subcutaneously in female immunodeficient mice. Tumor weight was assessed at the indicated days after injection. Values are means ± SD from 8 mice per group.

A Western blotting analysis revealed that the expression levels of the class II PI3K isoform PI3K-C2β increased in a panel of breast cancer cell lines compared to epithelial mammary breast cells (BRE80, HBL 100 and MCF10A), suggesting that PI3K-C2β expression is upregulated in breast cancer cell lines compared to ‘normal’, immortalized breast cells (Figure 1A). To investigate the role of PI3K-C2β, we downregulated the levels of the enzyme in human breast cancer cell lines MCF7, T47D and MDA-MB-231 using a specific shRNA (Figure 1B, 1C). Stable cells containing a control, non-targeting shRNA (“sh scrambled”) were also generated (Figure 1B, 1C). Western blot analyses confirmed inhibition of PI3K-C2β protein expression in all cell lines (Figure 1B, 1C). Specifically a strong reduction in PI3K-C2β levels was obtained in one clone of MCF7 cells (clone 1, Figure 1B), which was subsequently used for all studies performed in MCF7 cells. Similarly, expression of PI3K-C2β was strongly inhibited in T47D and MDA-MB-231 cell populations expressing the specific shRNA (Figure 1C). Importantly, no effect on the levels of the class I PI3K isoforms p110α, p110β and the class II enzyme PI3K-C2α was detected in these cells upon downregulation of PI3K-C2β (Figure 1C).

We next investigated the effect of PI3K-C2β downregulation on cell growth. Anchorage-independent growth assessed by soft agar assay was significantly reduced in T47D and MCF7 cells lacking PI3K-C2β (Figure 1D), indicating that this enzyme is required for 3D growth of these breast cancer cell lines. To evaluate the role of PI3K-C2β *in vivo*, control MCF7 cells (sh scrambled) and MCF7 lacking PI3K-C2β (sh PI3K-C2β) were injected orthotopically into the mammary fat pad of nude mice treated with estrogen pellets. Data indicated that downregulation of PI3K-C2β significantly reduced tumors volume measured at the end of the experiments (day 51 after implant, Figure 1E). We then injected sh scrambled and sh PI3K-C2β MDA-MB-231 cells in the mammary fat pad of nude mice and followed tumors growth. These data clearly suggested a reduced tumor growth in mice bearing shPI3K-C2β MDA-MB-231 cells compared to mice injected with control cells, although differences did not reach statistical significance possibly because of high variability of tumors volume (Figure 1F). Specifically, at the last day of the experiment (73 days following implant), the weight of tumors generated by sh PI3K-C2β MDA-MB-231 was 55% lower than tumors generated by control cells (Figure 1F). On the other hand growth curves were almost overlapping when the two stable cell lines were implanted subcutaneously in mice, suggesting a potential role for the tumor microenvironment in PI3K-C2β-dependent breast cancer development and progression (Figure 1G).

Taken together these data reveal a key role for PI3K-C2β in breast cancer growth *in vitro* and *in vivo*, especially in an estrogen-dependent *in vivo* xenograft.

### PI3K-C2β regulates breast cancer cell proliferation *in vitro* and cell cycle progression

**Figure 2:**
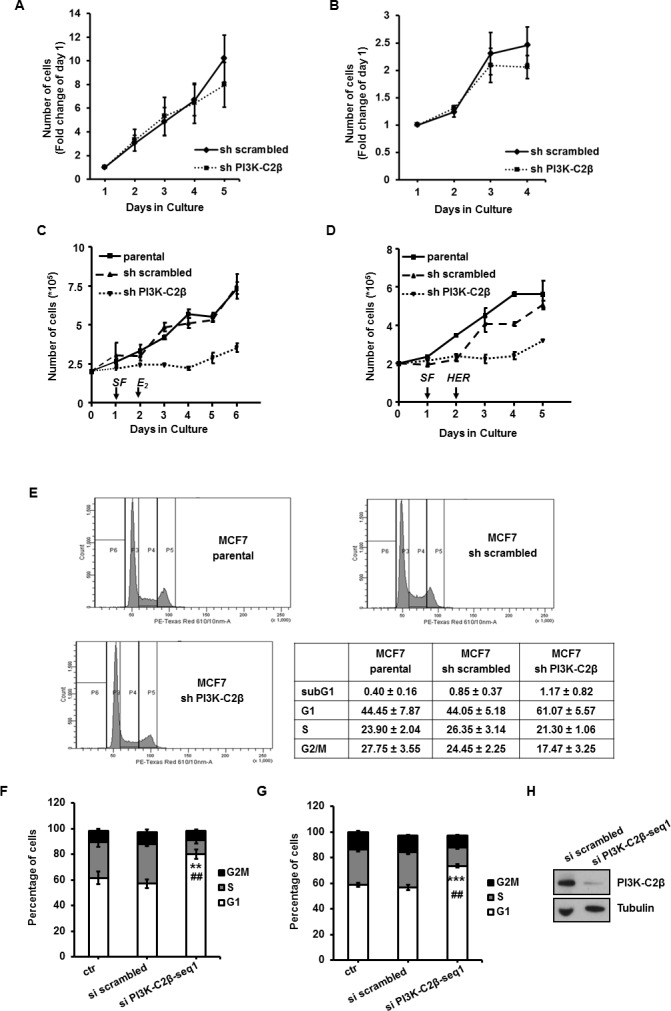
PI3K-C2β regulates breast cancer cell proliferation and cell cycle progression **A.**, **B.** The indicated T47D (A) and MCF7 (B) cells were incubated in normal growing media and counted at the indicated days. Data are means ± s.e.m. from *n* = 4 (A) and *n* = 3 (B, except for day 4 *n* = 2) independent experiments. **C.**, **D.** The indicated MCF7 cell lines were plated in 6 well plates. After 24h cells were incubated in phenol red-free/serum free (SF) medium for further 24h before incubation in phenol red-free/serum free media containing 10nM E_2_ or 50ng/ml HER. Cell growth was assessed by cell counting at the indicated days. Data are means ± s.e.m. from at least *n* = 3 independent experiments. **E.** Results from cell cycle analysis performed by FACS in the indicated cell lines. Data are means ± s.e.m. from at least *n* = 3 independent experiments. **F.** sh scrambled MCF7 cells were transfected with a non targeting siRNA (si scrambled), siRNA specifically targeting PI3K-C2β or transfection reagent alone (control, ctr). The following day cells were incubated with phenol red-free/serum-free media for 24h and then incubated for further 24h with growing media. Data indicate results from FACS analysis from *n* = 4 independent experiments (apart from si scrambled, *n* = 3). **p = 0.0028 *vs* si scrambled (*t*-Test, one tailed distribution, two sample, unequal variance) ##*p* = 0.0084 *vs* control (*t*-Test, one tailed distribution, paired). **G.** sh scrambled MCF7 cells were transfected and starved as in F and then incubated for 48h with growing media. Data indicate results from FACS analysis from *n* = 5 independent experiments. ***p = 0.00056 *vs* si scrambled, ##*p* = 0.0013 *vs* control (*t*-Test, one tailed distribution, paired). **H.** Representative blot of PI3K-C2β levels in lysates from sh scrambled MCF7 cells transfected with non targeting siRNA (si scrambled) and a specific siRNA targeting PI3K-C2β. Tubulin was used as loading control.

To better investigate the specific role of PI3K-C2β in breast cancer cell growth, we assessed the effect of its downregulation in different experimental conditions. Counting of cells in culture incubated in growing media [containing phenol red and 10% fetal bovine serum (FBS)] indicated that growth of T47D (Figure 2A) and MCF7 (Figure 2B) cells at early passages was not impaired upon downregulation of PI3K-C2β. On the other hand, when MCF7 cells were starved in phenol red-free/serum-free media for 24h and then stimulated with phenol red/serum free media supplemented with 17β-Oestradiol (E_2_)- or heregulin B1 (HER), a clear inhibition of cell proliferation was detected in MCF7 lacking PI3K-C2β (Figure 2C, 2D). No difference was observed between parental cells and sh scrambled MCF7 (Figure 2C, 2D). Similarly, cell proliferation induced by HER (Supplementary Figure S1A) and E_2_ (Supplementary Figure S1B) was impaired in sh PI3K-C2β T47D cells compared to control cells.

To further investigate the specific role of PI3K-C2β, MCF7 cells were synchronized by incubation in phenol red-free/serum-free media for 24h followed by incubation in growing media (Figure 2E). Cell cycle analysis indicated that in these experimental conditions downregulation of PI3K-C2β increased the percentage of cells in the G1 phase while reducing the percentage of cells in the G2/M phase of the cell cycle (Figure 2E). No difference was detected between sh scrambled and parental cells (Figure 2E). Similar results were obtained when sh scrambled MCF7 were transiently transfected with a specific siRNA targeting PI3K-C2β, incubated in phenol red-free/serum-free media for 24h and then in growing media for further 24h (Figure 2F) or 48h (Figure 2G). Efficient downregulation of PI3K-C2β was confirmed by Western blot (Figure 2H).

These data indicate that PI3K-C2β regulates HER- and E_2_-induced cell proliferation and cell cycle progression.

### PI3K-C2β regulates cyclin B1 expression

**Figure 3:**
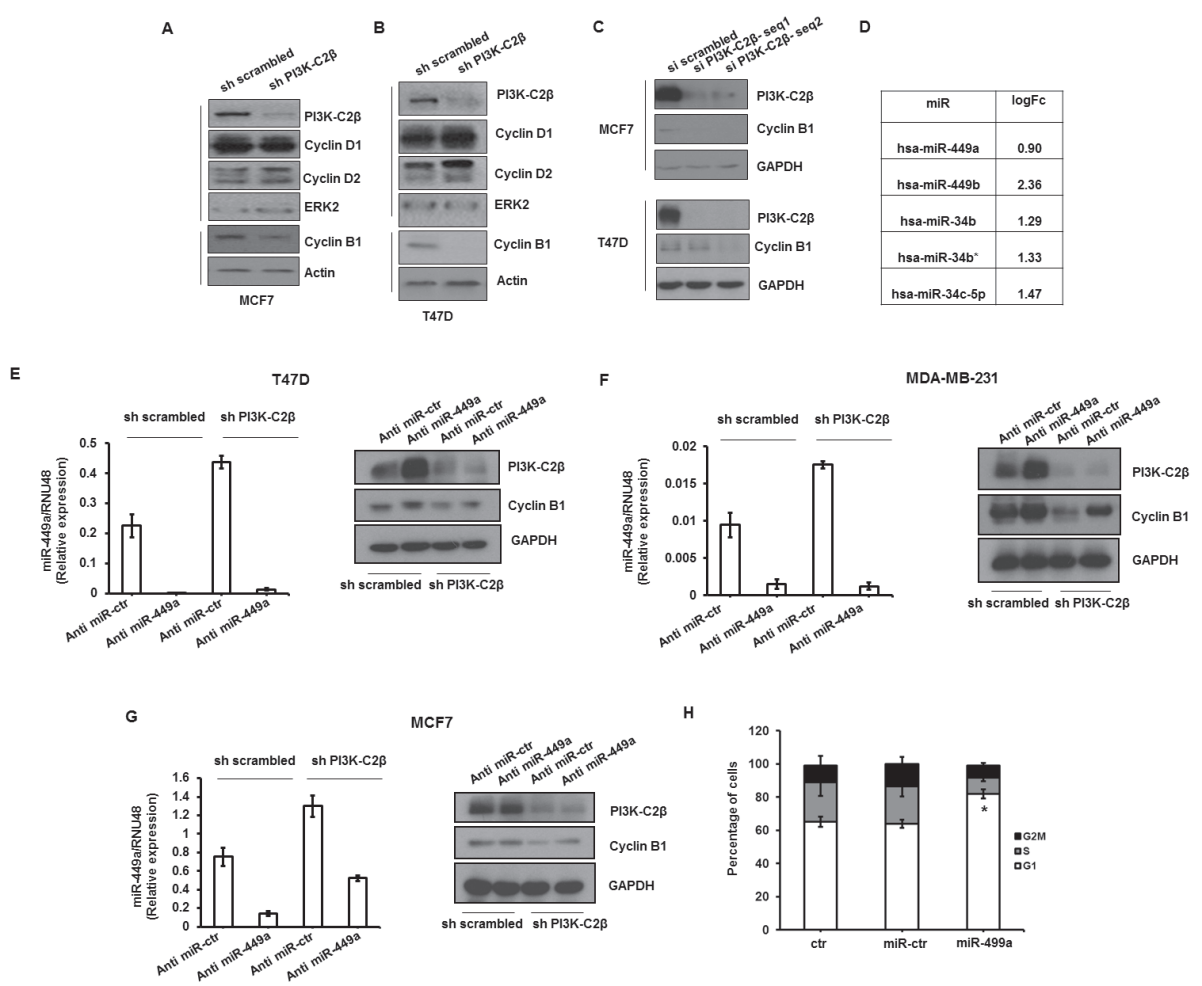
PI3K-C2β regulates cyclin B1 expression through modulation of miR-449a **A.**, **B.** Protein expression levels of cyclin D1, cyclin D2 and cyclin B1 in the indicated stable MCF7 and T47D cell lines assessed by Western blotting analysis. Downregulation of PI3K-C2β was also confirmed in these lysates. ERK2 and Actin were used as loading control. **C.** MCF7 and T47D were transiently transfected with the indicated siRNAs. Efficient downregulation of PI3K-C2β as well as expression levels of cyclin B1 were assessed by Western blotting at 72 h post transfection. GAPDH was used as loading control. **D.** Log-fold change (LogFc) values of the indicated miRs as assessed by miRs Array (Agilent platform) showing increased levels in T47D cells lacking PI3K-C2β compared to control, sh scrambled cells. **E.**-**G.** The indicated cell lines were transfected with a specific anti miR-449a or a non targeting anti miR (anti miR-ctr). Graphs show levels of miR-449a in the corresponding stable cells at 72h following transfection with the indicated anti miRs. Data shown are means ± ± s.e.m. from at least *n* = 3 independent experiments. Representative Western blots show the protein levels of PI3K-C2β and cyclin B1 in the corresponding cells. GAPDH was used as loading control. **H.** sh scrambled MCF7 cells were transfected with mimic miR449a (100nM), negative control miR (miR-ctr) or transfection reagent alone (control). Cells were then incubated with phenol red-free/serum free medium for 24h before incubation in growing medium for 24h. Data from FACS analysis are from *n* = 3 independent experiments (except for control *n* = 2). *p = 0.037 *vs* si scrambled (*t*-Test, one tailed distribution, paired).

To gain further insight into the mechanisms of the PI3K-C2β-dependent regulation of cell proliferation and cell cycle we then analyzed the activation of several signaling molecules involved in these processes. No effect on the activation of several components of the “classical” class I PI3K pathway, including Akt (Supplementary Figure S2A), 3-phosphoinositide dependent protein kinase 1 (PDK1), and the downstream effectors glycogen synthase kinase (GSK3)β (Supplementary Figure S2B), mechanistic target of rapamycin (mTOR) and S6 kinase (S6K) (Supplementary Figure S2C) was detected in MCF7 and T47D cells upon PI3K-C2β downregulation. Importantly, we observed that HER- and insulin-like growth factor-1 (IGF-1)-induced phosphorylation of PDK1, Akt, S6K, GSK3β and ERK1/2 was not affected by PI3K-C2β downregulation in MCF7 and T47D (Supplementary Figure S2D). Similarly, downregulation of PI3K-C2β in these cells did not impair Akt and ERK phosphorylation upon stimulation with insulin, HER and E_2_ (Supplementary Figure S2E). These data indicate that the reduced proliferation of sh PI3K-C2β MCF7 and T47D upon HER or E_2_ stimulation is not due to impairment in the activation of MAPK or classical PI3K/Akt pathways. On the other hand, Western blot analysis indicated that downregulation of PI3K-C2β strongly reduced the protein expression levels of cyclin B1 in MCF7 (Figure 3A) and T47D cells (Figure 3B). The effect was specific for cyclin B1 since no reduction in the expression levels of cyclin D1 and cyclin D2 was detected in these cells (Figure 3A, 3B). Reduced levels of cyclin B1 were also observed in MCF7 and T47D upon transient downregulation of PI3K-C2β using two distinct siRNAs (Figure 3C), ruling out any potential aspecific effect due to the chronic PI3K-C2β downregulation. Furthermore, cyclin B1 but not cyclin D1 protein levels, were also reduced in stable MDA-MB-231 lacking PI3K-C2β (Supplementary Figure S3A).

Taken together these data indicate that PI3K-C2β regulates cyclin B1 expression levels in breast cancer cells.

### PI3K-C2β regulates cyclin B1 *via* miR-449 regulation

We next sought to assess the mechanism by which PI3K-C2β regulates cyclin B1 expression. The observation that cyclin B1 mRNA levels were not altered in cells lacking PI3K-C2β compared to control cells (Supplementary Figure S3B), led us to hypothesize a potential role for microRNAs (miRs) in the PI3K-C2β-dependent regulation of cyclin B1 expression. To test this hypothesis, we first performed miR expression profiling in the stable T47D cell lines. This analysis revealed a selective upregulation of 16 miRs in T47D cells lacking PI3K-C2β compared to control cells, including 5 miRs belonging to the same family: miR-449a, miR-449b, miR-34b, miR-34b*, miR-34c-5p (Figure 3D). Interestingly, members of the miR-34 family have been shown to regulate cyclin B1 levels [20, 21] therefore, they could potentially be involved in the PI3K-C2β-mediated regulation of cyclin B1. To investigate this hypothesis, we first analyzed miRs levels in T47D stable cell lines by q-PCR to validate the results of the array. This analysis not only confirmed a very significant increase in the levels of miR-449a upon PI3K-C2β downregulation, but it also indicated that the levels of this specific miR were much higher than the levels of the other miRs investigated (Supplementary Figure S3C). Upregulation of miR-34b (Supplementary Figure S3D), miR-34c (Supplementary Figure S3E) and miR-449b (Supplementary Figure S3F) was also confirmed in T47D lacking PI3K-C2β compared to control cells, although the levels of these miRs were much lower compared to the levels of miR449-a. Taken together these data indicated that PI3K-C2β regulates miR levels, in particular it down modulates miR-449a levels.

To determine whether the PI3K-C2β-mediated regulation of miR-449a was associated with modulation of cyclin B1 protein expression, we then analyzed the effect of miR-449a downregulation on cyclin B1 expression. Transfection of T47D (Figure 3E), MDA-MB-231 (Figure 3F) and MCF7 (Figure 3G) with a specific anti miR-449a successfully reduced the levels of miR-449a in all cell lines (graphs in Figure 3E-3G). More importantly, transfection with the specific anti-miR-449a resulted in increased cyclin B1 protein expression, in particular in cells lacking PI3K-C2β (Figure 3E-3G). In addition, transfection of sh scrambled MCF7 cells with miR-449a increased the percentage of cells in G1 phase of the cell cycle assessed upon starvation in phenol red-free/serum-free media and incubation in growing media for 24h (Figure 3H), consistent with data obtained upon PI3K-C2β downregulation (Figure 2E-2H).

These data indicate that downregulation of PI3K-C2β reduces cyclin B1 levels through upregulation of miR-449a and that miR-449a is involved in the regulation of cell cycle progression.

### miR-449a levels are downregulated in primary human breast cancer samples

To determine whether modulation of miR-449a levels occurs in human breast cancer specimens, we analyzed two independent datasets of primary human breast cancers, publicly available on Array Express. As shown in Supplementary Table S1, with reference to the E-GEOD-19783 dataset, we observed that the expression of miR-449a was significantly downregulated in basal compared to LUM A and normal-like samples (logFC −0.9 and −0.4, respectively, *p*-value < 0.01), and in TP53 mutated samples *versus* TP53 wild-type ones (logFC −0.5, *p*-value < 0.01). Analysis of the E-GEOD-12848 dataset confirmed the downregulation of miR-449a (Supplementary Table S1) in basal-like samples compared to LUM A ones, and in TP53 mutated *versus* TP53 wild-type cancers. Additional information on tumor grade revealed that the expression of miR-449a was significantly repressed in grade 3 tumors when compared to grade 1-2 tumors. A similar down-regulation, although not statistically significant, was observed for miR-449a in T3-T4 samples *versus* T1 ones (Supplementary Table S1). Taken together these data indicate that miR-449a is downregulated in human breast cancer tissues and negatively associated to aggressiveness/progression.

### β catenin is involved in miR-449a regulation in MDA-MB-231 cells

**Figure 4:**
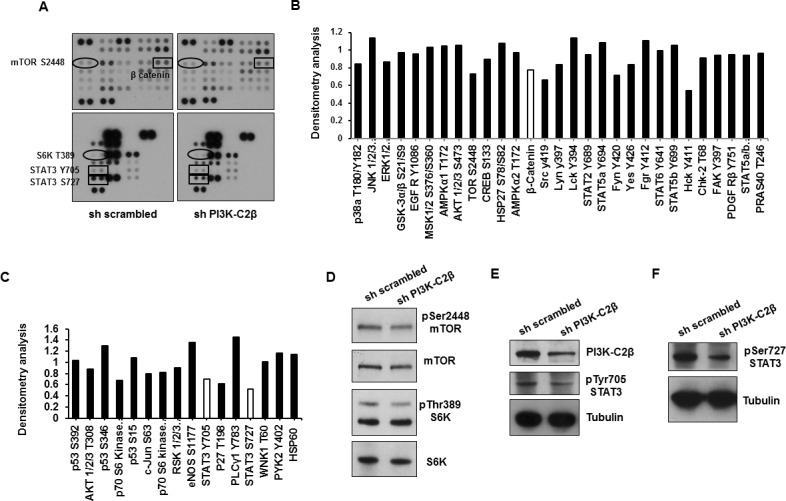
PI3K-C2β modulates phosphorylation of specific proteins **A.** Images from Proteome Profiler Human Phospho-Kinase Array assay performed in stable MDA-MB-231 cells kept in serum. **B.**,**C.** Densitometry analysis of the phospho array. For each protein data show values from sh PI3K-C2β samples expressed as fold change compared to corresponding values from sh scrambled samples. **D.**Western blotting analysis of mTOR and S6K phosphorylation at their residues Ser2448 and Thr389 respectively. Membranes were then stripped and re-incubated with anti mTOR and anti S6K antibodies. **E.**,**F.** Western blotting analysis of STAT3 phosphorylation at its residues Tyr705 and Ser727. Tubulin was used as loading control.

**Figure 5:**
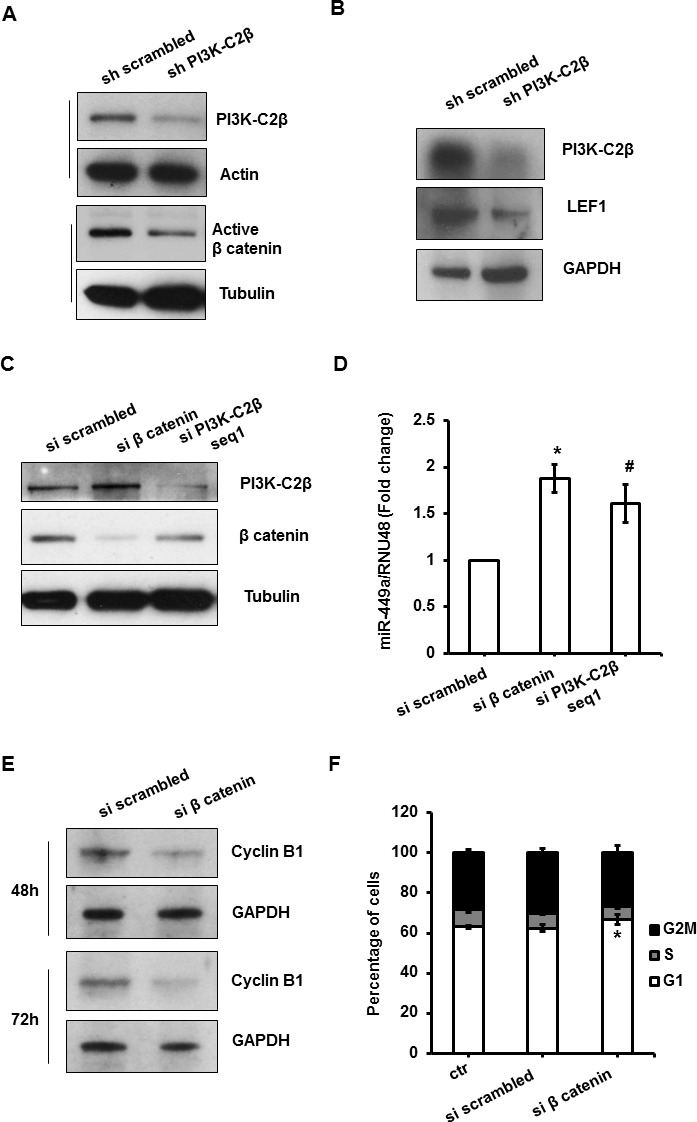
PI3K-C2β regulates miR449a/cyclinB1 through modulation of LEF1/β catenin pathway in MDA-MB-231 cells **A.**,**B.** Western blotting analysis of active β catenin (A) and LEF1 protein levels (B) in the indicated MDA-MB-231 stable cell lines. Tubulin was used as loading control and levels of PI3K-C2β were also assessed. **C.**, **D.** MDA-MB-231 cells were transiently transfected with specific siRNAs targeting β catenin and PI3K-C2β respectively. A non targeting (si scrambled) siRNA was used as control. Efficient downregulation of the proteins was determined after 48h. Graph shows the effect of transient transfection with the indicated siRNAs on miR-449a levels. Data are expressed as fold change over miR-449a/RNU48 values in cells transfected with non targeting siRNA and are means ± s.e.m. of *n* = 4 (si scrambled, si β catenin) and n = 3 (si PI3K-C2β) independent experiments. *p = 0.012, #*p* = 0.026 (*t*-Test, one tailed distribution, paired). **E.** Western blotting analysis of cyclin B1 protein levels in MDA-MB-231 at 48h and 72h following transfection with the indicated siRNAs. **F.** MDA-MB-231 cells were transiently transfected as before. Cells were incubated for 24h in phenol red-free/serum-free medium before further incubation for 24h in growing medium. Data are means ± s.e.m. of *n* = 3 independent experiments. *p = 0.024 (*t*-Test, one tailed distribution, paired).

To gain further insight into the signaling pathways involved in the PI3K-C2β-dependent regulation of miR-449a and cyclin B1, we then performed a phosphokinase array, monitoring phosphorylation and activation of ~50 proteins in the stable MDA-MB-231 cell lines (Figure 4A-4C). Densitometry analysis indicated that downregulation of PI3K-C2β selectively affected phosphorylation/activation of specific proteins (Figure 4B, 4C). Western blotting analysis to validate some of the phosphoarray results revealed only a partial inhibition of mTOR phosphorylation at Ser2448 and no inhibition of S6K phosphorylation at Thr389 in MDA-MB-231 upon downregulation of PI3K-C2β (Figure 4D), consistent with data obtained in MCF7 and T47D (Supplementary Figure S2C). The transcription factor Signal transducer and activator of transcription 3 (STAT3) was amongst the proteins whose phosphorylation was reduced in sh PI3K-C2β MDA-MB-231 compared to control cells, according to the results from the phosphoarray (Figure 4C). Further Western blot analysis confirmed that phosphorylation of STAT3 at both Tyr705 and Ser727 was indeed inhibited upon PI3K-C2β downregulation in MDA-MB-231 cells (Figure 4E, 4F). Analysis of the phosphoarray further revealed a specific downregulation of β catenin in shPI3K-C2β MDA-MB-231 (Figure 4B). Validation of the phosphoarray by Western blotting analysis confirmed reduced levels of active β catenin in MDA-MB-231 lacking PI3K-C2β (Figure 5A). To determine whether the PI3K-C2β-dependent regulation of β catenin was involved in the regulation of miR-449a and cyclin B1, TRANSFAC was used to identify transcription factors with putative binding sites within 10K bases upstream the miR-449a TSS. Factors with either core match and matrix match equal 1 were further selected. 90 putative binding sites were found with core match and matrix match equal 1, 35 of which had negative orientation (as miR-449a). Among those 35, 5 corresponded to the transcription factor Lymphoid enhancer-binding factor-1 (LEF1). Since T-cell factor /LEF transcription factors are responsible for the vast majority of β catenin signaling outputs [22, 23], we then determined whether PI3K-C2β was also involved in LEF1 regulation. Western blotting analysis indicated that LEF1 protein expression levels were indeed downregulated in MDA-MB-231 lacking PI3K-C2β (Figure 5B), suggesting that PI3K-C2β could regulate miR499a by modulating the β catenin/LEF1 pathway. To investigate this hypothesis, we next determined the effect of β catenin downregulation on miR-449a levels. Transient downregulation of both β catenin and PI3K-C2β in MDA-MB-231 (Figure 5C) significantly increased the levels of miR-449a (Figure 5D). Consistent with the observed upregulation of miR-499a, transient downregulation of β catenin reduced cyclin B1 protein levels in MDA-MB-231 (Figure 5E), as observed in cells lacking PI3K-C2β (Supplementary Figure S3A). Furthermore, downregulation of β catenin in sh scrambled MDA-MB-231 induced a very slight, albeit significant increase in the percentage of cells in G1 phase of the cell cycle assessed upon starvation in phenol red-free/serum-free media followed by incubation with growing media for 24h (Figure 5F).

Taken together these data indicate that β catenin is involved in the PI3K-C2β-dependent regulation of miR-449a and cyclin B1 levels in MDA-MB-231 cells.

It must be noted that downregulation of β catenin (Supplementary Figure S4A) did not affect cell cycle progression in sh scrambled MCF7 cells, assessed following 24h (Supplementary Figure S4B) or 48h (Supplementary Figure S4C) reintroduction of growing media. This would be consistent with reported data indicating a role for β catenin in MDA-MB-231 but not in MCF7 cells [24]. We therefore investigated whether STAT3 was involved in the regulation of cell cycle in sh scrambled MCF7 cells. Data showed that downregulation of STAT3 in these cells (Supplementary Figure S4D) did not impair cell cycle progression assessed by FACS analysis upon 24h starvation in phenol red-free/serum-free media followed by 24h (Supplementary Figure S4E) or 48h (Supplementary Figure S4F) incubation in growing media. These data indicate that STAT3 does not play a major role in the PI3K-C2β-dependent mechanism of cell cycle progression in MCF7 cells.

### PI3K-C2β regulates senescence *via* miR-449 regulation

It has been recently shown that miR-449 induces cell senescence in prostate and gastric cancer cells [25, 26]. Interestingly, we observed increased cell size in both MCF7 (Supplementary Figure S5A) and T47D (Supplementary Figure S5B) lacking PI3K-C2β. Increased levels of the senescence marker β-galactosidase were also observed in both cell lines upon PI3K-C2β downregulation (Supplementary Figure S5C) and reduced levels of SIRT1 were detected in MCF7 lacking PI3K-C2β (Supplementary Figure S5D), suggesting that PI3K-C2β may have a role in senescence regulation. To determine whether this PI3K-C2β-mediated regulation of cellular senescence was dependent on miR-449a regulation, we next investigated the effect of miR-449a downregulation on SIRT1 levels. Data showed that downregulation of miR-449a (performed in parallel with experiments presented in Figure 3G) was indeed able to counteract the effect of PI3K-C2β downregulation on SIRT1 levels and to increase SIRT1 protein levels (Supplementary Figure S5D). Furthermore, analysis of cellular proliferation by cell counting showed growth impairment in MCF7 and T47D lacking PI3K-C2β when kept in culture for more than 10 passages (Supplementary Figure S5E, S5F).

### PI3K-C2β regulates cell invasion and *in vivo* metastasis formation

**Figure 6:**
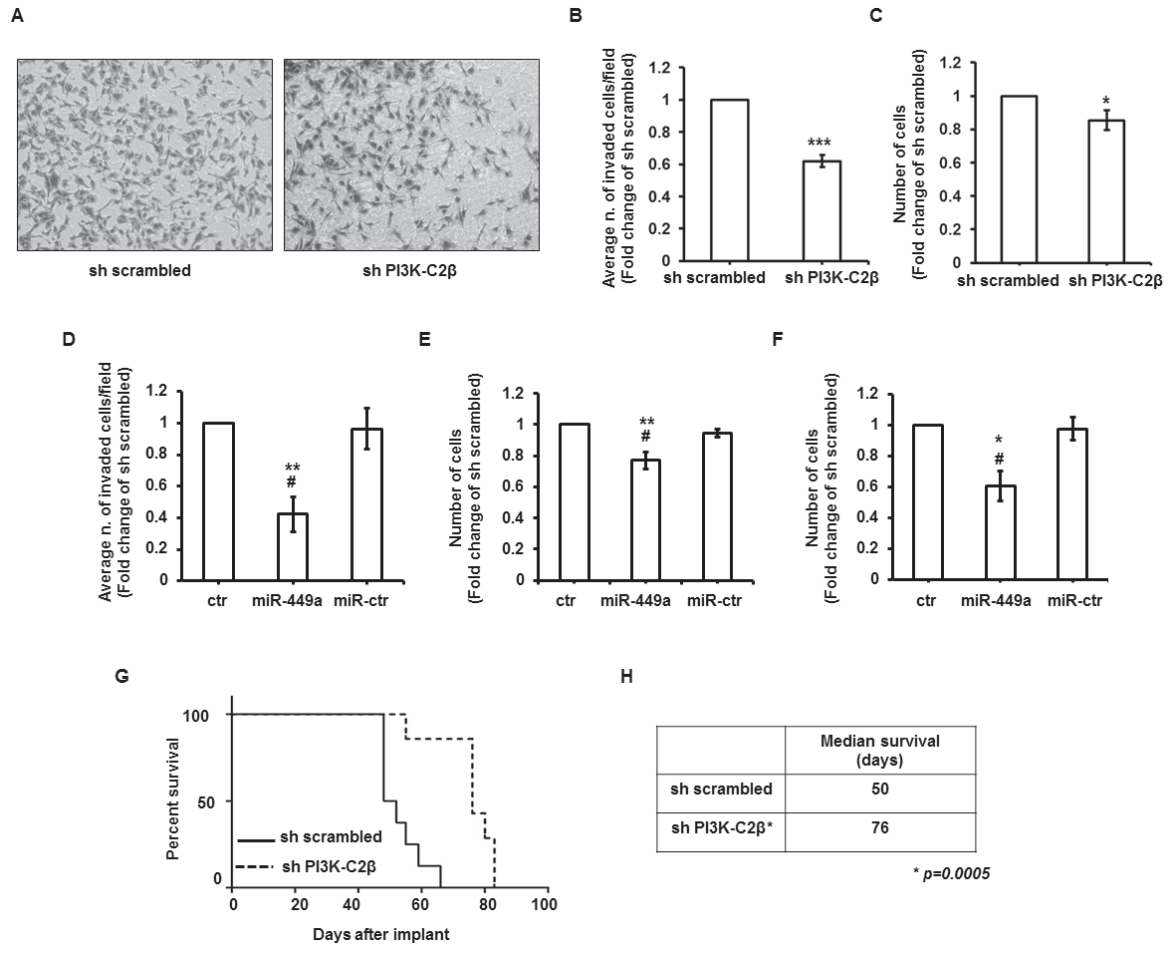
PI3K-C2β regulates cell invasion and metastasis formation **A.**, **B.** Representative images and results from invasion assays performed in the indicated stable MDA-MB-231 cell lines. Graph indicates the number of invaded cells/field expressed as fold increase over control (sh scrambled cells) and are means ± s.e.m. from *n* = 8 independent experiments. ***p = 8.71*10^−6^ (*t*-Test, one tailed distribution, paired). In these experiments the average number of invaded cells/field was 345±79 (sh scrambled) and 214±52 (sh PI3K-C2β). **C.** Cells were plated as for invasion assays, serum starved overnight and then re-plated for further 48h. Data are expressed as fold change over control (sh scrambled cells) and are means ± s.e.m. from *n* = 6 independent experiments. *p = 0.029 (*t*-Test, one tailed distribution, paired). **D.** sh scrambled MDA-MB-231 cells were transfected with mimic miR-449a, negative control miR (miR-ctr) or transfection reagent alone (control, ctr). The following day cells were starved overnight and then detached for invasion assay, as described in the Methods. Graph indicates the number of invaded cells/field expressed as fold increase over control cells and are means ± s.e.m. from *n* = 5 independent experiments (except for miR-ctr, *n* = 4). **p = 0.0031 *vs* control (*t*-Test, one tailed distribution, paired), #*p* = 0.029 *vs* miR-ctr (*t*-Test, one tailed distribution, two sample unequal variance). In these experiments the average number of invaded cells/field was 445±48 (control), 207±80 (miR-449a) and 429±80 (miR-ctr). **E.** sh scrambled MDA-MB-231 cells were transfected and starved as in D. Results from cell counting performed at the start of invasion assays, i.e. 48h after transfection. Data are expressed as fold change over control cells and are means ± s.e.m. from *n* = 5 independent experiments. **p = 0.0067 *vs* control, #*p* = 0.015 *vs* miR-ctr (*t*-Test, one tailed distribution, paired). **F.** sh scrambled MDA-MB-231 cells were transfected and starved as in D. Results from cell counting performed at the end of the invasion assays. Data are expressed as fold change over control cells and are means ± s.e.m. from *n* = 5 independent experiments (except for miR-449a, *n* = 4).*p = 0.013 *vs* control, #*p* = 0.035 *vs* miR-ctr (*t*-Test, one tailed distribution, two samples unequal variance). **G.**, **H.** Survival curve of mice injected intravenously with the indicated stable MDA-MB-231 cell lines. Each group consisted of 8 mice. The median survival in days for the two groups is also shown. The statistical difference in survival (*p* = 0.0005) was calculated using the Log-rank test.

Data so far indicated that PI3K-C2β has a key role in breast cancer growth both *in vivo* and *in vitro*. Since PI3K-C2β has previously been involved in cancer cell migration [16, 17], we next investigated the potential role of PI3K-C2β in cell invasion and metastasis using the highly invasive breast cancer cell line MDA-MB-231. As shown in Figure 6A, 6B, stable PI3K-C2β downregulation indeed affected MDA-MB-231 cell invasion. The effect was primarily due to a defect in invasion, as counting of cells plated in parallel and incubated in 10% FBS for 48h revealed only a slight reduction in cell number (Figure 6C). Since it was previously reported that β catenin plays a role in MDA-MB-231 cell migration [27] and our data indicated that both PI3K-C2β and β catenin regulate miR-449a levels, we then investigated whether miR-449a was also involved in cell invasion. Transfection of sh scrambled MDA-MB-231 cells with mimic miR-449a significantly reduced cell invasion whereas a control miR had no effect (Figure 6D), indicating a role for the PI3K-C2β-dependent regulation of miR-449a in invasion of MDA-MB-231 cells. To assess whether cell proliferation was also affected in the same experimental conditions, transfected cells were starved overnight, detached and counted (Figure 6E). Cells were then re-plated in growing media and counted after 48h (i.e. the duration of the invasion assay, Figure 6F). These data showed that transfection of mimic miR-449a reduced the number of cells (Figure 6E, 6F). However, the fact that downregulation of PI3K-C2β did not strongly affect cell number in the same experimental conditions (Figure 6C) possibly suggests additional roles for miR-449a in MDA-MB-231 cells. On the other hand, we cannot exclude the possibility that the difference may be due to different amounts of miR-449a present in sh PI3K-C2β cells and in sh scrambled transiently transfected with the mimic miR-449a. Nevertheless, data indicate that both PI3K-C2β and miR-449a are involved in MDA-MB-231 cell invasion.

We then investigated the effect of PI3K-C2β downregulation in a lung metastasis model *in vivo*. Data indicated that survival of mice injected with sh PI3K-C2β MDA-MB-231 cells was significantly increased compared to mice injected with sh scrambled cells (Figure 6G, 6H). Specifically, we observed that only one mouse out of 8 mice injected with sh scrambled MDA-MB-231 cells was alive at day 59 after implant (Figure 6G). In contrast, almost all mice (7 out of 8) injected with MDA-MB-231 lacking PI3K-C2β were alive at day 59 after implant. The difference in survival between the two groups of mice was statistically significant (*p* = 0.0005). These data suggest that PI3K-C2β has a key role in breast cancer metastasis formation.

To directly investigate the potential role of PI3K-C2β in metastasis formation, mice injected with sh scrambled or sh PI3K-C2β MDA-MB-231 cells were sacrificed at the same time point to determine the number and size of lung metastases and to evaluate the tumor burden of metastasis, as described in the Methods. Images of the excised lungs are presented in Supplementary Figure S6A. First we observed that at the chosen time point only 2 out of 8 mice injected with sh PI3K-C2β MDA-MB-231 cells had developed lung metastases compared to 4 out of 7 mice injected with sh scrambled cells (Supplementary Table S2 and S3). Importantly, the number of metastases (Supplementary Figure S6B) and the metastasis burden (Supplementary Figure S6C) were clearly reduced in mice injected with cells lacking PI3K-C2β, compared to mice injected with control cells. A detailed analysis of number/size of metastases in each mouse and metastasis burden is presented in Supplementary Table S2 and S3.

These data indicate that PI3K-C2β is required for breast cancer metastasis formation.

### Expression of PI3K-C2β in human breast tissues

**Figure 7:**
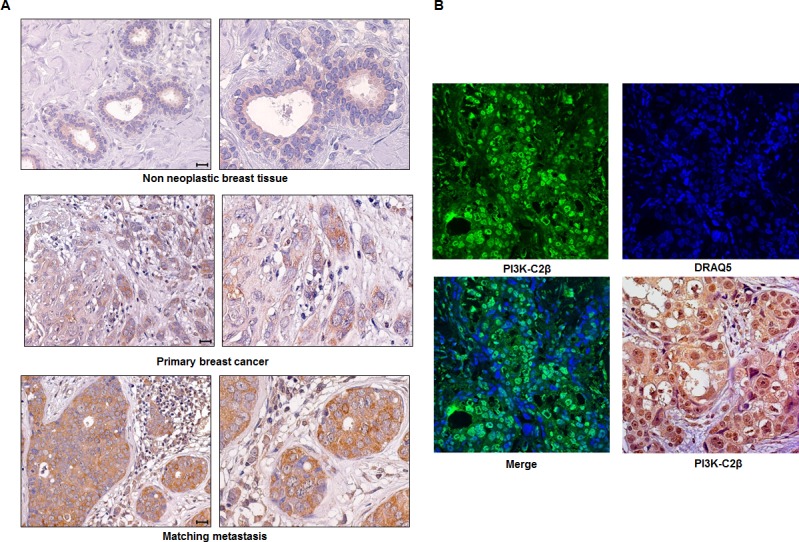
Expression of PI3K-C2β in breast tissues **A.** Representative staining pattern of PI3K-C2β expression in TDLU from a non-neoplastic breast tissue, a primary breast cancer and matching metastasis. Panels on the right show magnifications of images presented in the corresponding left panels. Scale bars = 20μm. **B.** Confocal microscopy images of PI3K-C2β (green signal), DRAQ5 (blue signal), and their merged signal in a breast cancer tissue. This case shows colocalization of PI3K-C2β expression with DRAQ5 nuclear dye. Bright field microscopy image of PI3K-C2β expression on the same case is also shown (original magnification 40x).

Once determined the key role of PI3K-C2β in breast cancer cells *in vitro* and in *in vivo,* we next investigated the expression of this enzyme in human non-neoplastic breast tissues, primary breast tumors and lymph-node metastases. Representative staining patterns of PI3K-C2β expression are shown in Figure 7. Whole sections of non-neoplastic breast tissues, stained with the anti PI3K-C2β antibody, revealed a very weak expression of the enzyme in the cytoplasm of luminal epithelia of terminal duct lobular units (TDLU) and of galactoforous ducts, but not in myoepithelia (Figure 7A). On the other hand, PI3K-C2β was clearly detectable in 45 out of 90 (50.0%) primary breast cancer specimens (Figure 7A), with the proportion of tumor cells positive for cytoplasmic PI3K-C2β being in the range of 5 to 100% (49.3±5.0, mean±SE). A nuclear specific staining was also seen in a smaller cell proportion (16.0±6.6, mean±SE). These cells also exhibited concomitant cytoplasmic expression of PI3K-C2β. Confocal microscopy analysis confirmed the nuclear localization of PI3K-C2β initially observed by bright field microscopy (Figure 7B). Spearman's correlation (Table 1) indicated that the expression of PI3K-C2β was directly correlated with the proliferative activity, as assessed by Ki-67 labeling index (*P* = 0.002), and with cyclin B1 expression (*P* < 0.001).

**Table:** Spearman's correlation among the indicated markers assessed in primary breast tumors (*n*=90)

	ER	PgR	Ki-67	PI3K-C2β	Cyclin B1	β catenin	pSTAT3 (Y705)
**ER**							
**Rho** **P**	1	**0.545** **<0.001**	−0.121 0.255	0.042 0.696	−0.059 0.581	−0.003 0.980	0.127 0.325
**PgR**							
**Rho** **P**	**0.545** **<0.001**	1	**−0.259** **0.014**	−0.127 0.234	**−0.223** **0.036**	**0.296** **0.007**	0.008 0.953
**Ki-67**							
**Rho** **P**	−0.121 0.255	**−0.259** **0.014**	1	**0.330** **0.002**	**0.447** **<0.001**	−0.111 0.321	0.010 0.941
**PI3K-C2β**							
**Rho** **P**	0.042 0.696	−0.127 0.234	**0.330** **0.002**	1	**0.368** **<0.001**	−0.038 0.735	0.187 0.146
**Cyclin B1**							
**Rho** **P**	−0.059 0.581	**−0.223** **0.036**	**0.447** **<0.001**	**0.368** **<0.001**	1	−0.076 0.503	0.023 0.860
**β catenin**							
**Rho** **P**	−0.003 0.980	**0.296** **0.007**	−0.111 0.321	−0.038 0.735	−0.076 0.503	1	−**0.266** **0.040**
**pSTAT3 (Y705)**							
**Rho** **P**	0.127 0.325	0.008 0.953	0.010 0.941	0.187 0.146	0.023 0.860	−**0.266** **0.040**	1

Rho = Spearman's coefficient correlation. Significant correlations (*P* < 0.05) are indicated in bold.

When PI3K-C2β expression was analyzed in twenty primary tumors/metastases pairs, we observed that the percentages of PI3K-C2β positive tumor cells in the synchronous lymph-node breast cancer metastases ranged from 71 to 100, with a mean±SE of 90.0±1.6, whereas the mean±SE percentage of cells positive for PI3K-C2β in the corresponding primary tumors was 47.9±9.0. These data indicated that metastatic tumors contained statistically significantly higher numbers of PI3K-C2β positive cells (*P* < 0.001 by independent samples *t*-test).

Taken together these data indicate that PI3K-C2β is barely detectable in non-neoplastic breast tissues, whereas it is clearly expressed in breast primary tumors where it correlates with proliferative activity. Furthermore data indicate that PI3K-C2β is highly expressed in lymph-node breast cancer metastases.

PI3Ks have been firmly established as key players in the development and progression of many cancer types and indeed PI3K inhibitors have been developed and are currently being tested in clinical trials [4]. One of the limitations in our understanding of the contribution of PI3Ks to cancer progression comes from the fact that the majority of the studies have been selectively focused on the involvement of one specific isoform, p110α, which is mutated in several cancer types [28]. The potential contribution of the other PI3K isoforms to cancer development and progression has been mostly disregarded. Only recently an increasing interest in understanding the role of the distinct PI3K isoforms has emerged [7, 29, 30]. Based on accumulating evidence suggesting that members of the class II subgroup of PI3Ks can have a role in cancer [7], here we investigated the potential role of the class II isoform PI3K-C2β in breast cancer. We detected accumulation of this specific isoform in breast cancer cell lines and human breast cancer specimens, suggesting a specific role for PI3K-C2β in this cancer type. A search in the cBio cancer genomics portal [31, 32] indicated that alteration of *PIK3C2B* has been observed by sequencing and copy number analysis of breast cancer specimens. Specifically both amplification (Supplementary Figure S7A) and point mutations (Supplementary Figure S7A, S7B) have been detected. Importantly, Kaplan-Meier curves for invasive breast cancer cases indicated reduced survival in patients with *PIK3C2B* alteration (Supplementary Figure S7C, S7D). These data support the conclusion that PI3K-C2β has a key role in breast cancer progression and metastasis. Nevertheless, to the best of our knowledge our study is the first to actually report an increased protein expression of PI3K-C2β in breast cancer cell lines, primary breast tumors and corresponding lymph-node metastases.

Our data show that PI3K-C2β is required for breast cancer growth both *in vitro* and *in vivo*. In particular, data suggest that this enzyme may have a key role in proliferation induced by growth factors, such as estrogen and heregulin that are critical for breast cancer progression. Indeed the estrogen-induced *in vivo* tumor growth was inhibited upon orthotopic implantation of sh PI3K-C2β MCF7 compared to sh scrambled MCF7 cells. A trend towards inhibition of tumor growth was also observed upon implantation of sh PI3K-C2β MDA-MB-231 in the mammary fat pad, whereas no difference was observed when these cells were implanted subcutaneously, possibly suggesting a role for the microenvironment in PI3K-C2β-dependent tumor progression. Consistent with a role for PI3K-C2β in breast cancer growth, a significant correlation between PI3K-C2β and Ki-67 proliferation index was observed in human breast cancer specimens.

In an effort to identify the mechanisms of the specific PI3K-C2β-dependent regulation of cell growth, we observed that key signaling pathways were not altered in breast cancer cells upon specific downregulation of PI3K-C2β. In particular, no difference in Akt activation was detected, consistent with our previous observations that class II PI3Ks activate distinct signaling pathways compared to the well-known class I PI3K/Akt pathway [7]. In contrast, we observed that downregulation of PI3K-C2β reduced cyclin B1 protein expression. Interestingly, cyclin B1 expression is known to increase during the G2 phase of the cell cycle [33] when PI3K-C2β is most active [34] supporting the conclusion that PI3K-C2β is involved in regulation of cyclin B1 levels. On the other hand, it is worth mentioning that a previous study detected cyclin B1 in the G1 phase in cells from breast cancer tissues [35], which would be consistent with our observation that downregulation of PI3K-C2β increases the percentage of cells in G1 phase following cellular synchronization. Importantly, cyclin B1 protein expression is upregulated in many breast cell lines and human mammary tumors [33]. Furthermore, cyclin B1 has been found to associate with poor prognosis and to be a predictor and classifier of outcomes in breast cancer [36, 37].

We further show that PI3K-C2β-dependent regulation of cyclin B1 occurs through modulation of the specific microRNA miR-449a. Specifically, we demonstrate that downregulation of PI3K-C2β increases miR-449a levels. To the best of our knowledge this is the first demonstration of a role for a class II PI3K isoform in miR regulation. miR-449a belongs to the miR-34 family, which is expressed at a low level in several cancer cell lines and solid tumors including breast cancer [38]. More specifically, previous analysis of miRNA expression in 101 tumor samples from breast cancer patients indicated that miR-449a was downregulated in highly proliferative samples, with negative correlation with the cell cycle genes [38]. Downregulation of miR-449a in breast cancer was also confirmed by our analysis of publicly available databases. Reduced proliferation [36] and inhibition of the BrdU proliferation index [39] was observed in MCF7 transfected with miR-449a. In particular, these cells appeared to be completely arrested in G1 phase in the presence of nocodazole [39]. Consistent with these data, we observed that transfection of MCF7 cells with mimic miR-449a was able to induce G1 arrest, similarly to the effect observed upon downregulation of PI3K-C2β.

The specific targets of miR-449 have only been partially identified and cyclin B1 is indeed among the validated miR-449 targets [25]. Consistent with this, our data demonstrate that counteracting the upregulation of miR-449a induced by PI3K-C2β downregulation, using specific anti miR-449a increases cyclin B1 protein levels.

Our data further identified one of the mechanisms by which PI3K-C2β can potentially regulate the levels of miR-449a. Specifically, we observed that downregulation of PI3K-C2β was associated with reduced activation of β catenin in MDA-MB-231 cells and that downregulation of β catenin resulted in increased miR-449a levels. These data suggest that PI3K-C2β may regulate miR-449a in a mechanism involving β catenin activation in MDA-MB-231 cells. An interesting possibility is that PI3K-C2β may specifically regulate the cancer stem cell fraction where Wnt signaling plays a major role and therefore the observed effect of β catenin/LEF1 in MDA-MB-231 cells may be due to a reduction in stem cells number. There is evidence that indicates an involvement of β catenin in miRNA repression [40, 41], even though the mechanism by which β catenin downregulates miR-449a in breast cancer is currently unknown. Importantly, it has been reported that β catenin plays a critical role in breast cancer development and tumorigenesis [42] and it can be a prognostic marker for breast cancer [43]. On the other hand, our data indicate that downregulation of β catenin was not able to increase the percentage of MCF7 cells in G1 phase of the cell cycle, as observed upon downregulation of PI3K-C2β or transfection with the mimic miR-449a. This is consistent with a previous study indicating that inhibition of β catenin/cAMP-responsive element-binding protein-binding protein-mediated transcription, inhibited cell proliferation in MDA-MB-231 but not in MCF7 cells [24]. These results suggest the existence of additional, β catenin-independent mechanisms by which PI3K-C2β modulates miR-449a levels in MCF7 cells. Since we observed that PI3K-C2β regulates activation of the transcription factor STAT3, which in turn can also be involved in miR-34 family regulation [44], we then tested whether STAT3 could be involved in PI3K-C2β-dependent regulation of miR-449a in MCF7 cells. However, the observation that downregulation of STAT3 did not affect cell cycle progression supports the conclusion that STAT3 is not involved in the regulation of miR-449a in MCF7 cells. While we cannot completely rule out the possibility that downregulation of either β catenin or STAT3 could possibly modulate miR-449a levels in MCF7 cells in different experimental conditions, overall these data suggest that PI3K-C2β modulates miR-449a levels in MCF7 cells through additional mechanisms of regulation. In this respect, it has been reported that miR-449a levels can be epigenetically repressed by histone H3 Lys27 trimethylation in MCF7 cells [39]. Furthermore, MCF7 ChIP-seq data from the ENCODE project revealed the presence of a H3K27me3 peak upstream miR-449a, whereas no activating histone modifications were detected (data not shown).

It has been further shown that miR-449a is able to inhibit migration and invasion and indeed a recent study reported that low expression of miR-449a was correlated with lymph-node metastasis [45]. These data indicated that miR-449 is a potential tumor suppressor that regulates cell cycle, invasion and metastasis by targeting multiple oncogenes. Consistent with this, we observed that downregulation of PI3K-C2β, as well as transfection of cells with mimic miR-449a, inhibited invasion of MDA-MB-231 cells. More importantly, we observed that survival of mice injected with sh PI3K-C2β MDA-MB-231 was significantly increased compared to mice injected with sh scrambled cells in an *in vivo* model of lung metastasis. Additional experiments revealed a reduced number of metastasis and reduced metastasis burden in mice injected with cells lacking PI3K-C2β compared to mice injected with control cells. These data strongly supported the conclusion that PI3K-C2β has a key role in metastasis formation induced by highly invasive breast cancer cells. Indeed, further analysis of the expression levels of PI3K-C2β in twenty primary tumors-metastasis pairs by immunohistochemistry analysis revealed that PI3K-C2β expression was significantly increased in lymph-node metastasis compared to matching primary tumors.

In summary, in this study we reported that PI3K-C2β is overexpressed in breast cancer cell lines and in human breast tumors and it has a critical role in the regulation of breast cancer cell growth. We demonstrated that downregulation of PI3K-C2β inhibits breast cancer cell invasion *in vitro* and breast cancer metastasis *in vivo*. Increased levels of PI3K-C2β were also detected in lymph-nodes metastasis compared to primary tumors. Data further indicated that PI3K-C2β regulates cyclin B1 expression through modulation of miR-449a. In addition, we showed that this novel PI3K-C2β/miR-449a pathway can also play a key role in the regulation of cell invasion. These data demonstrate that PI3K-C2β plays a pivotal role in breast cancer progression as well as in metastasis formation, and provide a strong rationale for the development of compounds that inhibit this PI3K isoform as novel potential therapeutic strategies.

## MATERIALS AND METHODS

### Chemicals and reagents

Human heregulin B1 (HER) and human insulin like growth factor-1 (IGF-1) were obtained from Peprotec EC Ltd. (London, UK). Human insulin (INS), human 17β-Oestradiol (E_2_), Protease inhibitor cocktail, Phosphatase inhibitor cocktail A and Phosphatase inhibitor cocktail B were from Sigma-Aldrich (Gillingham, UK). BCA Protein Assay kit was from Thermo Scientific Pierce (Thermo Fisher Scientific, Inc, Rockford, IL, USA). Anti PI3K-C2β, anti PI3K-C2α and mouse anti cyclin B1 antibodies were from BD Biosciences (Oxford, UK). Rabbit anti cyclin B1, anti cyclin D1, anti cyclin D2, rabbit anti phospho Thr202/Tyr204 ERK1/2, anti phospho Ser473 Akt, anti phospho Ser2448 mTOR, anti phospho Thr389 S6K, anti phospho Ser241 PDK1, anti phospho Ser9 GSK3β, anti phospho Tyr705 Stat3, anti phospho Ser727 STAT3, anti GSK3β, anti PDK1, anti S6K, anti mTOR, anti p110α, anti p110β, anti β catenin were from Cell Signaling Technology, Inc. (Danvers, MA, USA). Anti Akt, mouse anti phospho Thr202/Tyr204 ERK1/2, anti ERK2, anti actin, anti β tubulin, anti LEF-1 were from Santa Cruz Biotechnology, Inc. (Dallas, Texas, USA). Anti active β catenin (that recognises active form of β catenin, dephosphorylated on Ser37 or Thr41) was from Merck Millipore (Billerica, MA, USA). Anti GAPDH was from Abcam (Cambdrige, UK). Anti DRAQ5 was from BioStatus (Shepshed, Leicestershire, UK). Horseradish peroxidase-conjugated secondary anti-mouse, anti-rabbit and anti-goat antibodies were from DAKO UK Ltd. (Ely, UK) or from Sigma Aldrich (Gillingham, UK). Enhanced chemiluminescence reagent (ECL) was from GE Healthcare (Little Chalfont, UK) or Merck Millipore. Non targeting siRNA (“si scrambled”) was from Ambion^®^ (Life Technologies, Paisley, UK). siRNA targeting PI3K-C2β (sequence 1) was from Qiagen (Limburg, Netherlands), all other siRNAs were from Dharmacon, Inc. (GE Healthcare). Anti miR control and anti-miR-449a were from Life Technologies. Unless otherwise stated all other reagents were obtained from Sigma-Aldrich.

### Cell culture and transfection

The BRE80 cell line was a kind gift from Dr. Maria Konstantoulakis (University College London, UK). All other cell lines were obtained from American Type Culture Collection (ATCC Middlesex, UK). MCF10A and BRE80 cell lines were cultured in MEBM^®^ phenol red free media, supplemented with MEGM^®^ SingleQuots^®^ (Lonza, Walkersville MD). HBL-100, MDA-MB-231, MDA-MB-468, SKBR-3, MCF7 and T47D cell lines were all cultured in DMEM media supplemented with 10% FBS, penicillin/streptomycin, and L-glutamine, all from Life Technologies. All cell lines were maintained at 37°C in a humidified atmosphere of 95% air and 5% CO_2_. siRNAs and anti miRs were transfected using Oligofectamine™ (Life Technologies) or HiPerFect^®^ (Qiagen), according to the manufacturer's instructions.

### Generation of stable cell lines

Specific shRNA targeting PI3K-C2β was designed based on a previously validated sequence [16] and subcloned into pSuperior vector (Oligogene, Seattle, WA, USA). A recombinant pSuperior containing a control (scrambled) shRNA was also generated. MCF7 were transfected using the AMAXA nucleofector electroporation system (Lonza, Walkersville, MD, USA), following the manufacturer's standard operating instructions. T47D were transfected using Lipofectamine^®^ (Life Technologies) according to the manufacturer's instructions. Stable MDA-MB-231 cells were generated by retroviral infection, as previously described [46]. Single clones (MCF7) and cell populations (T47D, MDA-MB-231) were selected and maintained in standard media with 1μg/ml (MCF7, MDA-MB-231) or 0.5 μg/ml (T47D) puromycin.

### Cell growth assays

For cell counting assays, cells were plated into six well Nunc multiwell plates at a density of 2 × 10^5^/well for MCF7 cells and at 5 × 10^5^/well for T47D. After 24h cells were incubated in DMEM/F12-phenol red-free and serum -free media supplemented with penicillin/streptomycin, and L-glutamine for further 24h before incubation in phenol red-free and serum-free media containing 10nM E_2_ or 50ng/ml HER.

For anchorage-independent growth, a mix of 0.6% agarose in DMEM supplemented with 10% FBS was allowed to polymerize in a 6 well plate for 30 min. MCF7 or T47D cells were then resuspended in a mix of 0.3% agarose in DMEM supplemented with 10% FBS and plated on the 6 well plate. DMEM containing 10% FBS was added on top of the second agarose layer and replaced once a week. After 3 weeks, the 6 well plate was incubated with 0.0005% crystal violet in methanol overnight and the number of colonies was assessed by cell counting.

### Cell cycle analysis

MCF7 cells were synchronized by incubation for 24h in media deprived of serum and phenol red followed by 24h or 48h incubation in growing media. After washing with PBS, cells were detached, collected and centrifuged at 1,200 rpm for 5 minutes. Pelleted cells were fixed in ice-cold 70% ethanol and re-suspended in 500μl Vindellövs Propidium Iodide solution (50 mg/ml). Cells were then analyzed by flow cytometry collecting 20,000 events per sample using Fluorescence activated cell sorting (FACS) Diva software.

### Western blotting analysis of signaling molecules

Activation of signaling molecules was analyzed either in cells kept in serum or upon cellular stimulation. Where indicated cells were serum deprived for 24h in DMEM/F12-phenol red free (Life Technologies), supplemented with penicillin/streptomycin, and L-glutamine. Cells were washed with HEPES-buffered incubation medium (HBM: NaCl 140 mM, KCl 5 mM, NaHCO_3_ 5 mM, MgCl_2_, 1.1 mM, Na_2_HPO_4_ 1.2 mM, CaCl_2_ 1.2 mM, glucose 5.5 mM and HEPES 20 mM, pH 7.4) and incubated for 10 min with fresh HBM containing 10μg/ml INS, 50ng/ml HER, 10ng/ml IGF-1 or 10nM E_2_. HBM was removed, and plates quickly washed with ice-cold PBS, pH 7.4 (Ca^2+^-free supplemented with 200 μM EGTA) on ice. The cell monolayer was rapidly scraped and collected in ice-cold lysis buffer [50 mM Tris, pH 7.5, 150 mM NaCl, 1% Triton X-100, 2 mM EDTA, 2 mM EGTA, 1μl/ml Protease inhibitor cocktail, 1μl/ml Phosphatase inhibitor cocktail A and 1μl/ml Phosphatase inhibitor cocktail B] and left for 45 min. Samples were then centrifuged at 1000 × *g* for 5 min at +4°C to remove cell debris. The supernatant (crude homogenate) was kept, and protein concentration was determined by Bradford assay. Alternatively cells were lysed using 2% SDS in PBS. Samples were then boiled at 100°C for 5 min in SDS sample buffer [62.5mM Tris, 2% SDS, 10% glycerol, 5% β-mercaptoethanol and 0.<002% bromophenol blue]. Samples were separated by SDS-PAGE and transferred on nitrocellulose membranes by electroblotting. Membranes were then incubated in TBS (20 mM Tris, pH 7.5, 150 mM NaCl) or PBS supplemented with 0.1% (v/v) Tween 20 (TBS-T, PBS-T) and containing 5% skimmed milk powder for 1h at room temperature, followed by overnight incubation with primary antibodies at +4°C. After washing with TBS-T or PBS-T membranes were incubated with secondary antibodies for 1 h, washed with TBS-T or PBS-T and exposed to ECL reagent. When indicated membranes were stripped by washing twice for 10 min in TBST followed by two 30 min washes in glycine-based stripping buffer (1.5% glycine, 0.1% SDS, 1% Tween 20 pH 2.2). Alternatively membranes were stripped by incubation for 30 min at 50°C in SDS-based stripping buffer (62.5 mM Tris HCl pH 6.8, 0.5% SDS, 0.7% β mercapthoethanol).

### Human phosphokinase array

Analysis of the phosphorylation status of several kinases and proteins was performed using Proteome Profiler Human Phospho-Kinase Array Kit (Cat. No. ARY003, R&D Systems, Abingdon, UK) according to the manufacturer's instructions. Briefly, stable MDA-MB-231 cell lines kept in serum were washed in PBS and incubated with Lysis Buffer 6 for 30 min at +4°C. Cell debris were removed by centrifugation at 14,000 × g for 5 minutes and protein concentration was assessed by BCA Protein Assay. After blocking in Array Buffer 1 for 1h at room temperature each membrane was incubated with 600μg of cell lysate overnight at +4°C. Membranes were then washed with 1X Wash Buffer and incubated with diluted Detection Antibody Cocktail A and B for 2h at room temperature followed by incubation with Streptavidin-HRP for additional 30 min. Membranes were then incubated with Chemi Reagent Mix and exposed on an X-ray film for 1-10 minutes. Densitometry analysis was performed using Image J software according to the manufacturer's instructions.

### Invasion assay

Assay was performed in MDA-MB-231 stable cell lines as previously described [47].

### miRs analysis

To assess miRs levels cells were washed twice with PBS and lysed using QIAzol Lysis Reagent (Qiagen). Total RNA was extracted using miRNAse Mini Kit (Qiagen) and cDNA synthesised using TaqMan^®^ MicroRNA Reverse Transcription kit (Life Technologies) according to manufacturer's instructions. PCR was performed according to manufacturer's instructions (TaqMan^®^MicroRNA Assays Protocol, Life Technologies). Analysis of miRs expression was performed using the cycle threshold (Ct) and RNU48 as endogenous control. Levels of miRs were obtained using the ΔΔCt method and expressed as 2^(−ΔΔCt)^ values. All experiments were performed in triplicate.

### Immunohistochemistry

Tissue microarrays (TMA) were constructed by extracting 2-mm diameter cores of histologically confirmed invasive breast carcinoma areas from 90 invasive primary breast tumors, and matching lymph-node metastases (*n* = 20 pairs), as previously described [48]. TMA sections were stained using the monoclonal mouse anti-human PI3K-C2β at 1:500 dilution for 30 min after antigen retrieval performed by two microwave treatments at 750 W and 160W for 10 min each in 1M urea buffer (pH 8.0). Whole sections of non-neoplastic breast tissues from 10 patients were also stained. EnVision kit (Dako, Glostrup, Denmark) was used for signal amplification. After antigen retrieval by thermostatic bath at 96°C in 10 mM citrate buffer, pH 6.0 for 40 min sections were also incubated with anti-EstogenReceptor-α (ER) MoAb 6F11 (Menarini, Florence, Italy), anti-Progesteron Receptor (PgR) MoAb 1A6 (Menarini), and anti-Ki-67 MoAb MIB-1 (Dako) for 30 min at room temperature. For β -catenin (clone 14/β -catenin, BD Transduction Laboratories, San Jose, CA) epitope retrieval was done by microwave heated citrate buffer (pH 6.0), and in 1 mmol/L EDTA (pH 8.0) for pospho-Stat3 (Tyr705) (clone 3E2, Cell Signaling, Danvers, MA). The immunoreactions were revealed by a streptavidin-biotin-enhanced peroxidase system (Super Sensitive Link-Label IHC DetectionSystem; BioGenex, Space, Milan, Italy). For cyclin B1, staining was performed using a rabbit monoclonal anti-human antibody (clone Y106, Epitomics, Cambridge, UK), at 1:200 dilution for 30 min, after antigen retrieval performed by microwave treatment for 25 min in Tris-EDTA buffer (pH 9.0). An anti-rabbit EnVision kit (Dako) was used for signal amplification. Positive and negative controls were included for each antibody and in each batch of staining. Correlation between PI3K-C2β expression and ER, PgR, Ki-67, cyclin B1, β-catenin and pospho-Stat3 (Tyr705) expressions was assessed by Spearman's rho correlation. The independent samples *t*-test was used to compare the PI3K-C2β expression in primary tumors and matched lymph-node metastasis. The SPSS (version 15.0) statistical program (SPSS Inc., Chicago, IL) was used for analyses. All P values are two-sided; *p* < 0.05 was considered as statistically significant.

For confocal laser-scanning microscopy (LSM 510 META microscope, Zeiss, Jena, Germany), after antigen retrieval, sections were incubated with the mouse monoclonal antibody anti PI3K-C2β. Then, Alexa Fluor 488 conjugated rabbit anti-mouse IgG (Molecular Probes, Eugene, OR) was used (60 min incubation at 1:200 dilution) for signal detection. Cell nuclei were counterstained with DRAQ5 (BioStatus, Shepshed, Leicestershire, UK).

### *In vivo* experiments

Nude immunodeficient mice were obtained from Charles River, UK or Harlan-Italy and maintained under specific pathogen-free conditions with food and water provided ad libitum. All animals in this study were housed and sacrificed in accordance with Home Office Guidelines and under a project license. Procedures involving animals and their care were conducted in conformity with institutional guidelines that are in compliance with national (Legislative Decree 116 of January 27, 1992, Authorization n.169/94-A issued December 19, 1994, by Ministry of Health) and international laws and policies (EEC Council Directive 86/609, OJL 358. 1, December 12, 1987; Standards for the Care and Use of Laboratory Animals, United States National Research Council, Statement of Compliance A5023-01, November 6, 1998). Where required, mice were humanely sacrificed *via* a rising concentration of CO_2_ to near 100% followed by cervical dislocation.

For MCF7 orthotopic experiments cells were diluted in 200μl growth medium + 200μl BD Matrigel™ (BD Biosciences). The cell suspension was injected into the mammary fat pad of 5 week-old pathogen-free nude mice on day 0. E_2_ pellets (1.7mg/pellets, IRA, Sarasota, FL) were injected subcutaneously into the neck with pellet trochar (IRA). At the end of the experiment tumors volume was measured with caliper according to the formula length × width × height.

For experiments with MDA-MB-231 a total of 7 × 10^6^ exponentially growing cells were injected subcutaneously into the left flank of female 4-week-old athymic nude (nu/nu) mice. Eight mice/group were used in these experiments. For orthotopic tumor growth, mice were anesthetized with a continuous flow of 3% isoflurane/oxygen mixture. Then, a 1-cm incision in the skin closed to the mammary fat pad was performed to expose it, and 10^5^ cells in a total volume of 10 μl of PBS were injected into the mammary gland using an Hamilton^®^ syringe equipped with a 26-gauge needle. By exposing the fat pad, we were able to ensure that the cells were injected into the tissue and not into the s.c. space. Tumor diameters were measured with a caliper twice weekly until the animals were sacrificed. Tumor weight was derived from tumor volume (assuming a density of 1) by the formula: (length × width2)/2. Body weights were measured weekly.

To monitor survival in the lung metastasis *in vivo* experiment, 5 × 10^5^ cells were injected i.v. and the animals were monitored daily and body weights recorded. At symptoms of distress and body weight loss the animals were sacrificed and lungs excised. Survival of the animals was calculated from the day of cancer cells injection (day 0) to the day of sacrifice.

To monitor lung metastasis formation, 5 × 10^5^ cells were injected i.v. as above and mice were sacrificed the day after the first mouse from group injected with sh scrambled MDA-MB-231 cells died. Lungs were then excised and fixed in Bouin's solution (Bio-Optica). Superficial metastatic nodules were counted and measured using a dissecting microscope. Specifically number of colonies per lung and the diameter of colonies were recorded. Metastasis volume was calculated as diameter^3^(mm^3^)/2, and the lung tumor burden was derived from the sum of the volumes of all counted colonies, as previously reported [49].

### miRs array

miRs array was performed at the Genome Centre, Queen Mary University of London.

### miRs search

MetaCore^TM^ version 6.10 (GeneGo Inc) was used to identify miRs that were likely to target cyclin B1.

### Public datasets analysis

Two independent microarray datasets, publicly available on Array Express (E-GEOD-19783 and E-GEOD-12848), were analyzed for miR expression across 101 and 18 primary human breast cancer samples, respectively. Samples were stratified according to the grade, molecular tumor subtype (Lum A, Lum B, ERBB2+, Basal-like, Normal-like), Estrogen Receptor status, HER2 status, TP53 status, tumor size, cancer staging. For the E-GEOD-19783 dataset, raw data were available and the quantile algorithm was used for data normalization. For the E-GEOD-12848 dataset, the gTotalGeneSignal from GeneView files was used to analyze samples. Student *T*-test p-value and log base 2 fold change were calculated for each class comparison.

### TF binding site analysis

Match tool from TRANSFAC [50] was used for identification of transcription factors with putative binding sites within 10K bases upstream the miR-449a TSS. The weight matrix search was performed using a cut-off that minimizes the sum of both false positive and negative error rates.

### Cellular senescence assay

Cell senescence was assessed using the senescence β-galactosidase staining kit from Cell Signaling Technology, Inc. according to the manufacturer's instructions.

## SUPPLEMENTARY MATERIAL FIGURES AND TABLES



## References

[1] Higgins MJ, Baselga J (2011). Targeted therapies for breast cancer. The Journal of clinical investigation.

[2] Yuan TL, Cantley LC (2008). PI3K pathway alterations in cancer: variations on a theme. Oncogene.

[3] Wong KK, Engelman JA, Cantley LC (2010). Targeting the PI3K signaling pathway in cancer. Current opinion in genetics & development.

[4] Falasca M (2010). PI3K/Akt signalling pathway specific inhibitors: a novel strategy to sensitize cancer cells to anti-cancer drugs. Current pharmaceutical design.

[5] Vanhaesebroeck B, Guillermet-Guibert J, Graupera M, Bilanges B (2010). The emerging mechanisms of isoform-specific PI3K signalling. Nature reviews Molecular cell biology.

[6] Miller TW, Rexer BN, Garrett JT, Arteaga CL (2011). Mutations in the phosphatidylinositol 3-kinase pathway: role in tumor progression and therapeutic implications in breast cancer. Breast cancer research.

[7] Falasca M, Maffucci T (2012). Regulation and cellular functions of class II phosphoinositide 3-kinases. The Biochemical journal.

[8] Chiaretti S, Li X, Gentleman R, Vitale A, Wang KS, Mandelli F, Foa R, Ritz J (2005). Gene expression profiles of B-lineage adult acute lymphocytic leukemia reveal genetic patterns that identify lineage derivation and distinct mechanisms of transformation. Clinical cancer research.

[9] Qian Z, Fernald AA, Godley LA, Larson RA, Le Beau MM (2002). Expression profiling of CD34+ hematopoietic stem/progenitor cells reveals distinct subtypes of therapy-related acute myeloid leukemia. Proceedings of the National Academy of Sciences of the United States of America.

[10] Knobbe CB, Reifenberger G (2003). Genetic alterations and aberrant expression of genes related to the phosphatidyl-inositol-3′-kinase/protein kinase B (Akt) signal transduction pathway in glioblastomas. Brain pathology.

[11] Boller D, Doepfner KT, De Laurentiis A, Guerreiro AS, Marinov M, Shalaby T, Depledge P, Robson A, Saghir N, Hayakawa M, Kaizawa H, Koizumi T, Ohishi T, Fattet S, Delattre O, Schweri-Olac A (2012). Targeting PI3KC2beta impairs proliferation and survival in acute leukemia, brain tumours and neuroendocrine tumours. Anticancer research.

[12] Arcaro A, Khanzada UK, Vanhaesebroeck B, Tetley TD, Waterfield MD, Seckl MJ (2002). Two distinct phosphoinositide 3-kinases mediate polypeptide growth factor-stimulated PKB activation. The EMBO journal.

[13] Russo A, O'Bryan JP (2012). Intersectin 1 is required for neuroblastoma tumorigenesis. Oncogene.

[14] Koutros S, Schumacher FR, Hayes RB, Ma J, Huang WY, Albanes D, Canzian F, Chanock SJ, Crawford ED, Diver WR, Feigelson HS, Giovanucci E, Haiman CA, Henderson BE, Hunter DJ, Kaaks R (2010). Pooled analysis of phosphatidylinositol 3-kinase pathway variants and risk of prostate cancer. Cancer research.

[15] Liu P, Morrison C, Wang L, Xiong D, Vedell P, Cui P, Hua X, Ding F, Lu Y, James M, Ebben JD, Xu H, Adjei AA, Head K, Andrae JW, Tschannen MR (2012). Identification of somatic mutations in non-small cell lung carcinomas using whole-exome sequencing. Carcinogenesis.

[16] Maffucci T, Cooke FT, Foster FM, Traer CJ, Fry MJ, Falasca M (2005). Class II phosphoinositide 3-kinase defines a novel signaling pathway in cell migration. The Journal of cell biology.

[17] Katso RM, Pardo OE, Palamidessi A, Franz CM, Marinov M, De Laurentiis A, Downward J, Scita G, Ridley AJ, Waterfield MD, Arcaro A (2006). Phosphoinositide 3-Kinase C2beta regulates cytoskeletal organization and cell migration via Rac-dependent mechanisms. Molecular biology of the cell.

[18] Russo A, Okur MN, Bosland M, O'Bryan JP (2015). Phosphatidylinositol 3-kinase, class 2 beta (PI3KC2beta) isoform contributes to neuroblastoma tumorigenesis. Cancer letters.

[19] Arcaro A, Zvelebil MJ, Wallasch C, Ullrich A, Waterfield MD, Domin J (2000). Class II phosphoinositide 3-kinases are downstream targets of activated polypeptide growth factor receptors. Molecular and cellular biology.

[20] Bouhallier F, Allioli N, Lavial F, Chalmel F, Perrard MH, Durand P, Samarut J, Pain B, Rouault JP (2010). Role of miR-34c microRNA in the late steps of spermatogenesis. Rna.

[21] Marcet B, Chevalier B, Luxardi G, Coraux C, Zaragosi LE, Cibois M, Robbe-Sermesant K, Jolly T, Cardinaud B, Moreilhon C, Giovannini-Chami L, Nawrocki-Raby B, Birembaut P, Waldmann R, Kodjabachian L, Barbry P (2011). Control of vertebrate multiciliogenesis by miR-449 through direct repression of the Delta/Notch pathway. Nature cell biology.

[22] Gebeshuber CA, Sladecek S, Grunert S (2007). Beta-catenin/LEF-1 signalling in breast cancer--central players activated by a plethora of inputs. Cells, tissues, organs.

[23] Valenta T, Hausmann G, Basler K (2012). The many faces and functions of beta-catenin. The EMBO journal.

[24] Schade B, Lesurf R, Sanguin-Gendreau V, Bui T, Deblois G, O'Toole SA, Millar EK, Zardawi SJ, Lopez-Knowles E, Sutherland RL, Giguere V, Kahn M, Hallett M, Muller WJ (2013). beta-Catenin signaling is a critical event in ErbB2-mediated mammary tumor progression. Cancer research.

[25] Noonan EJ, Place RF, Basak S, Pookot D, Li LC (2010). miR-449a causes Rb-dependent cell cycle arrest and senescence in prostate cancer cells. Oncotarget.

[26] Bou Kheir T, Futoma-Kazmierczak E, Jacobsen A, Krogh A, Bardram L, Hother C, Gronbaek K, Federspiel B, Lund AH, Friis-Hansen L (2011). miR-449 inhibits cell proliferation and is down-regulated in gastric cancer. Molecular cancer.

[27] Dwyer MA, Joseph JD, Wade HE, Eaton ML, Kunder RS, Kazmin D, Chang CY, McDonnell DP (2010). WNT11 expression is induced by estrogen-related receptor alpha and beta-catenin and acts in an autocrine manner to increase cancer cell migration. Cancer research.

[28] Liu S, Knapp S, Ahmed AA (2014). The structural basis of PI3K cancer mutations: from mechanism to therapy. Cancer research.

[29] Falasca M, Maffucci T (2007). Role of class II phosphoinositide 3-kinase in cell signalling. Biochemical Society transactions.

[30] Maffucci T, Falasca M (2014). New insight into the intracellular roles of class II phosphoinositide 3-kinases. Biochemical Society transactions.

[31] Cerami E, Gao J, Dogrusoz U, Gross BE, Sumer SO, Aksoy BA, Jacobsen A, Byrne CJ, Heuer ML, Larsson E, Antipin Y, Reva B, Goldberg AP, Sander C, Schultz N (2012). The cBio cancer genomics portal: an open platform for exploring multidimensional cancer genomics data. Cancer discovery.

[32] Gao J, Aksoy BA, Dogrusoz U, Dresdner G, Gross B, Sumer SO, Sun Y, Jacobsen A, Sinha R, Larsson E, Cerami E, Sander C, Schultz N (2013). Integrative analysis of complex cancer genomics and clinical profiles using the cBioPortal. Science signaling.

[33] Kawamoto H, Koizumi H, Uchikoshi T (1997). Expression of the G2-M checkpoint regulators cyclin B1 and cdc2 in nonmalignant and malignant human breast lesions: immunocytochemical and quantitative image analyses. The American journal of pathology.

[34] Visnjic D, Curic J, Crljen V, Batinic D, Volinia S, Banfic H (2003). Nuclear phosphoinositide 3-kinase C2beta activation during G2/M phase of the cell cycle in HL-60 cells. Biochimica et biophysica acta.

[35] Shen M, Feng Y, Gao C, Tao D, Hu J, Reed E, Li QQ, Gong J (2004). Detection of cyclin b1 expression in g(1)-phase cancer cell lines and cancer tissues by postsorting Western blot analysis. Cancer research.

[36] Agarwal R, Gonzalez-Angulo AM, Myhre S, Carey M, Lee JS, Overgaard J, Alsner J, Stemke-Hale K, Lluch A, Neve RM, Kuo WL, Sorlie T, Sahin A, Valero V, Keyomarsi K, Gray JW (2009). Integrative analysis of cyclin protein levels identifies cyclin b1 as a classifier and predictor of outcomes in breast cancer. Clinical cancer research.

[37] Nimeus-Malmstrom E, Koliadi A, Ahlin C, Holmqvist M, Holmberg L, Amini RM, Jirstrom K, Warnberg F, Blomqvist C, Ferno M, Fjallskog ML (2010). Cyclin B1 is a prognostic proliferation marker with a high reproducibility in a population-based lymph node negative breast cancer cohort. International journal of cancer.

[38] Enerly E, Steinfeld I, Kleivi K, Leivonen SK, Aure MR, Russnes HG, Ronneberg JA, Johnsen H, Navon R, Rodland E, Makela R, Naume B, Perala M, Kallioniemi O, Kristensen VN, Yakhini Z (2011). miRNA-mRNA integrated analysis reveals roles for miRNAs in primary breast tumors. PloS one.

[39] Yang X, Feng M, Jiang X, Wu Z, Li Z, Aau M, Yu Q (2009). miR-449a and miR-449b are direct transcriptional targets of E2F1 and negatively regulate pRb-E2F1 activity through a feedback loop by targeting CDK6 and CDC25A. Genes & development.

[40] Ladeiro Y, Couchy G, Balabaud C, Bioulac-Sage P, Pelletier L, Rebouissou S, Zucman-Rossi J (2008). MicroRNA profiling in hepatocellular tumors is associated with clinical features and oncogene/tumor suppressor gene mutations. Hepatology.

[41] Huang K, Zhang JX, Han L, You YP, Jiang T, Pu PY, Kang CS (2010). MicroRNA roles in beta-catenin pathway. Molecular cancer.

[42] Wagh PK, Gray JK, Zinser GM, Vasiliauskas J, James L, Monga SP, Waltz SE (2011). beta-Catenin is required for Ron receptor-induced mammary tumorigenesis. Oncogene.

[43] Lin SY, Xia W, Wang JC, Kwong KY, Spohn B, Wen Y, Pestell RG, Hung MC (2000). Beta-catenin, a novel prognostic marker for breast cancer: its roles in cyclin D1 expression and cancer progression. Proceedings of the National Academy of Sciences of the United States of America.

[44] Rokavec M, Oner MG, Li H, Jackstadt R, Jiang L, Lodygin D, Kaller M, Horst D, Ziegler PK, Schwitalla S, Slotta-Huspenina J, Bader FG, Greten FR, Hermeking H (2014). IL-6R/STAT3/miR-34a feedback loop promotes EMT-mediated colorectal cancer invasion and metastasis. The Journal of clinical investigation.

[45] Luo W, Huang B, Li Z, Li H, Sun L, Zhang Q, Qiu X, Wang E (2013). MicroRNA-449a is downregulated in non-small cell lung cancer and inhibits migration and invasion by targeting c-Met. PloS one.

[46] Sala G, Dituri F, Raimondi C, Previdi S, Maffucci T, Mazzoletti M, Rossi C, Iezzi M, Lattanzio R, Piantelli M, Iacobelli S, Broggini M, Falasca M (2008). Phospholipase Cgamma1 is required for metastasis development and progression. Cancer research.

[47] Raimondi C, Chikh A, Wheeler AP, Maffucci T, Falasca M (2012). A novel regulatory mechanism links PLCgamma1 to PDK1. Journal of cell science.

[48] Lattanzio R, Marchisio M, La Sorda R, Tinari N, Falasca M, Alberti S, Miscia S, Ercolani C, Di Benedetto A, Perracchio L, Melucci E, Iacobelli S, Mottolese M, Natali PG, Piantelli M, Cinbo (2013). Overexpression of activated phospholipase Cgamma1 is a risk factor for distant metastases in T1-T2, N0 breast cancer patients undergoing adjuvant chemotherapy. International journal of cancer.

[49] Moschetta M, Pretto F, Berndt A, Galler K, Richter P, Bassi A, Oliva P, Micotti E, Valbusa G, Schwager K, Kaspar M, Trachsel E, Kosmehl H, Bani MR, Neri D, Giavazzi R (2012). Paclitaxel enhances therapeutic efficacy of the F8-IL2 immunocytokine to EDA-fibronectin-positive metastatic human melanoma xenografts. Cancer research.

[50] Kel AE, Gossling E, Reuter I, Cheremushkin E, Kel-Margoulis OV, Wingender E (2003). MATCH: A tool for searching transcription factor binding sites in DNA sequences. Nucleic acids research.

