# Supplementary material for: Class II phosphoinositide 3-kinase C2β regulates a novel signaling pathway involved in breast cancer progression
Source: Oncotarget. 2016 Feb 26;7(14):18325–45. doi: 10.18632/oncotarget.7761 (PMC4951291; doi:10.18632/oncotarget.7761)
Supplement: Supplementary file 1 [file oncotarget-07-18325-s001.pdf]

Class II phosphoinositide 3-kinase C2β regulates a novel signaling pathway involved in breast cancer progression

Supplementary Material

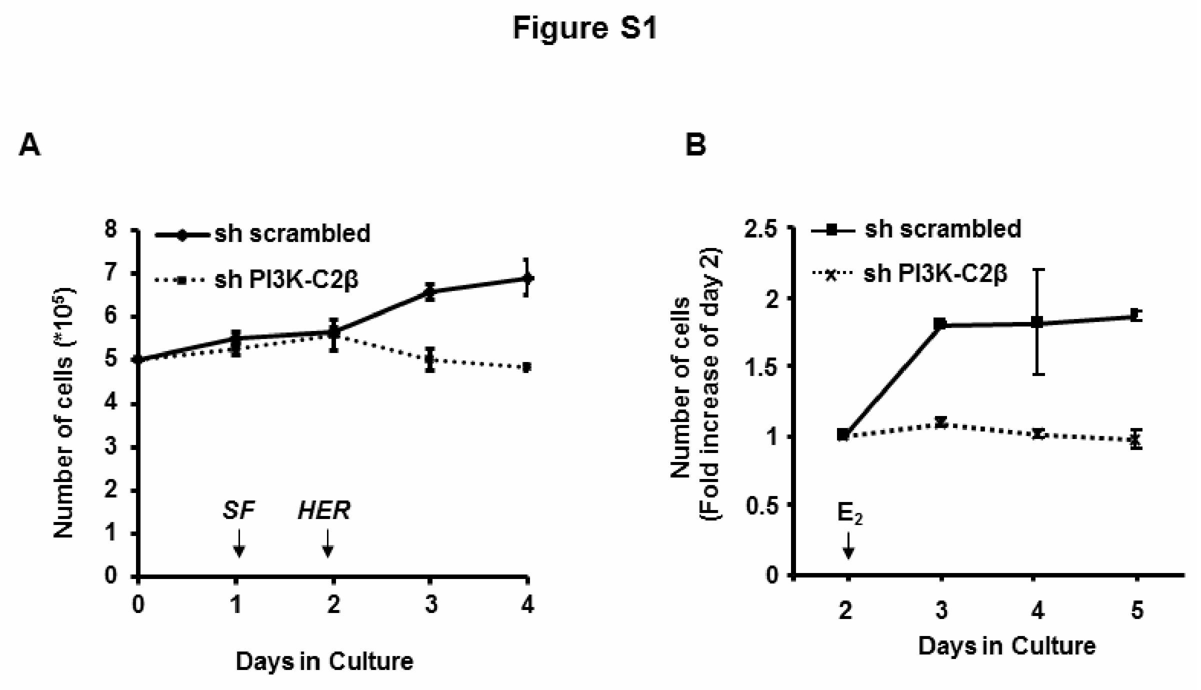

Figure S1: Effect of PI3K-C2β downregulation on HER- and E_2-dependent cell proliferation.

A,B The indicated T47D cell lines were plated in 6 well plates. After 24h cells were incubated in phenol red-free/serum-free media (SF) for further 24h before incubation in phenol red and serum free media containing 50ng/ml HER or 10nM E_2. Cell growth was assessed by cell counting at the indicated days. Data are means ± s.e.m. from at least n=3 independent experiments.

Figure S2

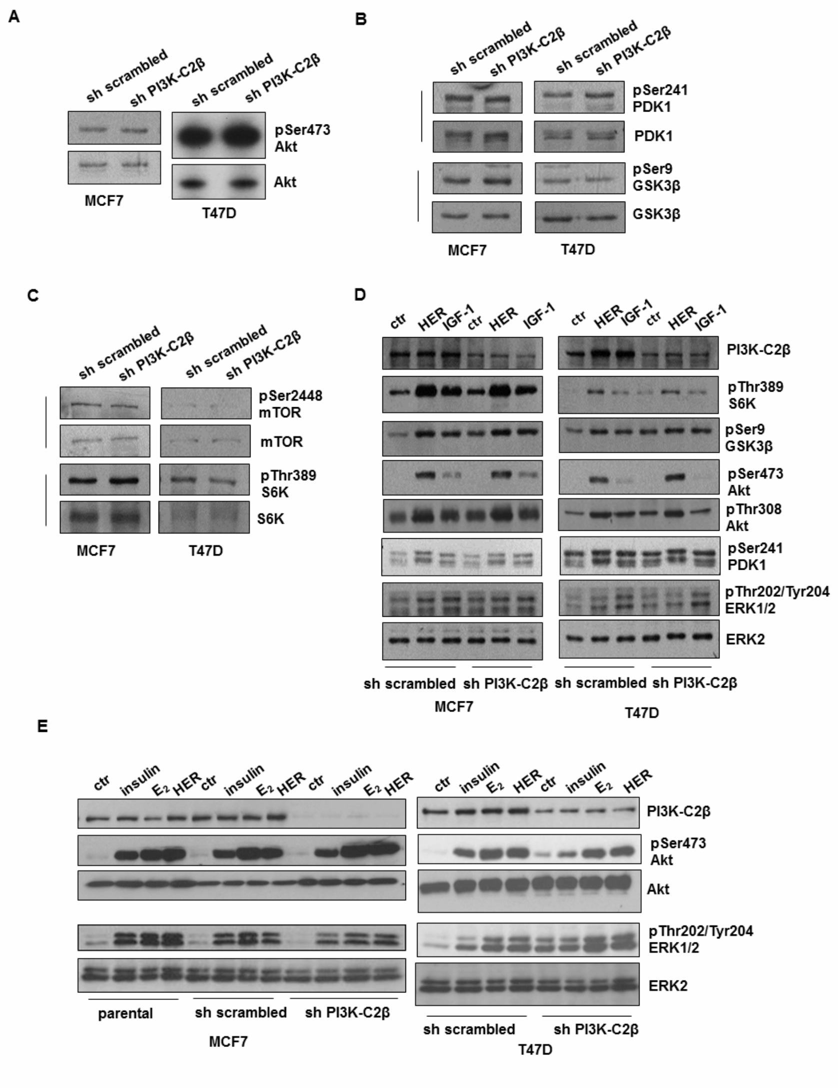

Figure S2: Effect of PI3K-C2β downregulation on signaling pathways.

A-C Western blot analysis of the indicated signaling molecules in stable MCF7 and T47D expressing or lacking PI3K-C2β. Membranes were first incubated with antibodies recognizing the phosphorylated form of the proteins, then stripped and re-incubated with antibodies recognizing the total proteins.

D,E Serum starved stable cell lines were stimulated with 10µg/ml INS, 50ng/ml HER, 10ng/ml IGF-1 or 10nM E_2 for 10mins. Phosphorylation of the indicated proteins was assessed by Western blotting. Membranes were then stripped and re-incubated with antibodies recognizing the total proteins.

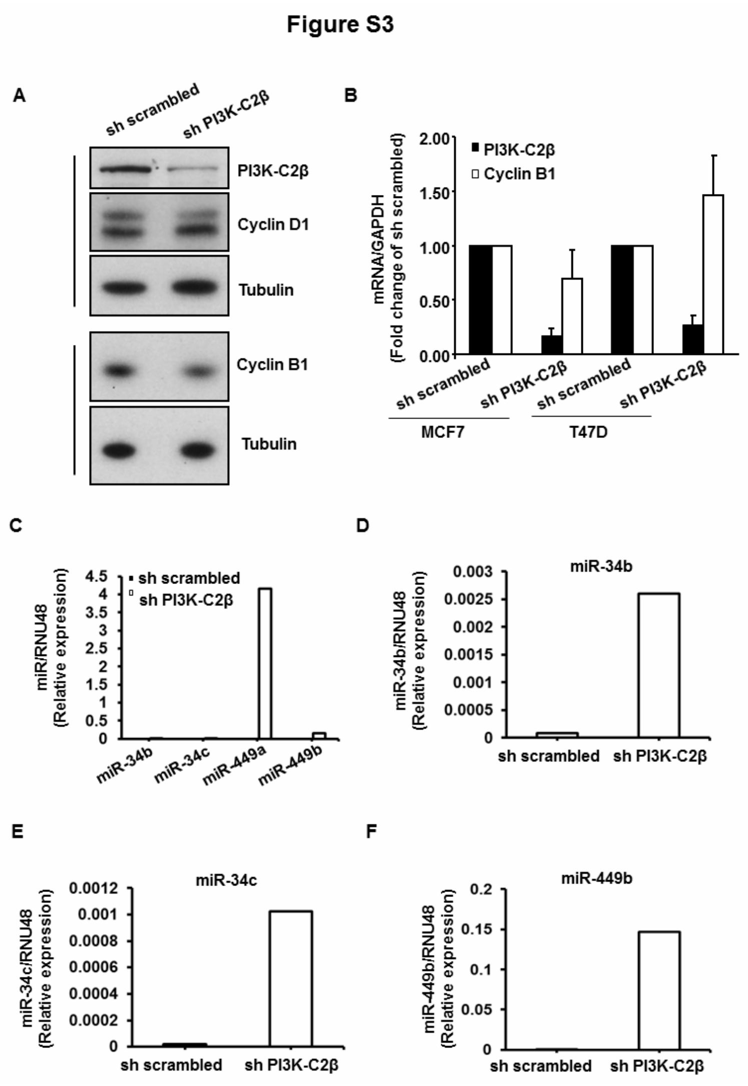

Figure S3: PI3K-C2β regulates cyclin B1 levels through miR-449a regulation.

A Representative Western blots showing the protein levels of PI3K-C2β, cyclin D1 and cyclin B1 in the indicated MDA-MB-231 cell lines. Tubulin was used as loading control.

B mRNA levels of cyclin B1 were assessed in the indicated MCF7 and T47D stable cell lines by qPCR. Reduced levels of PI3K-C2β mRNA in the knockdown cell lines were also confirmed.

C-F Levels of the indicated miRs were determined in the indicated T47D. Results from a representative analysis are presented.

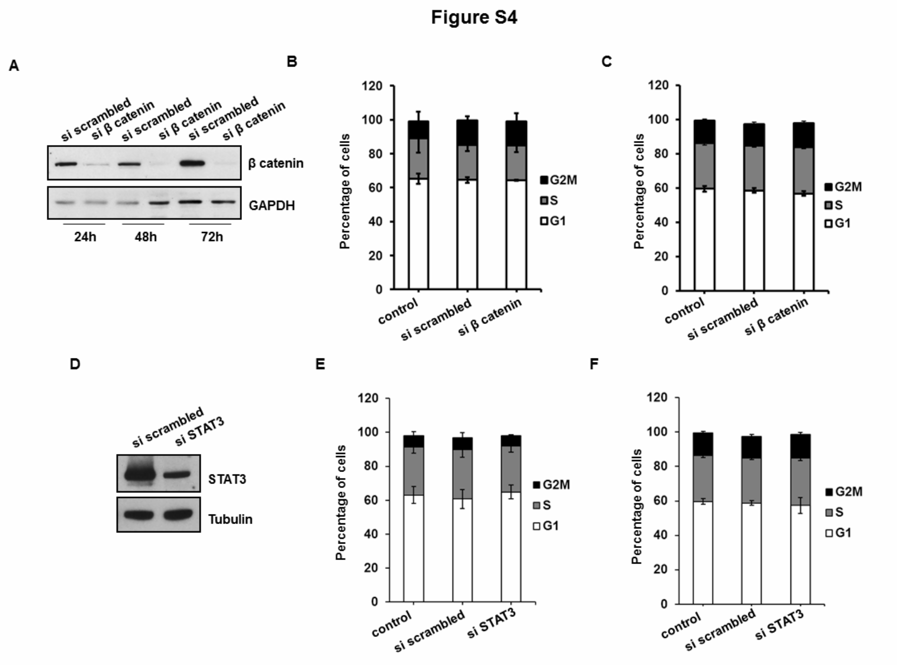

Figure S4: STAT3 and β catenin are not involved in regulation of cell cycle progression in MCF7 cells.

A-C sh scrambled MCF7 cells were transfected with siRNA specifically targeting β catenin, non targeting siRNA (si scrambled) or transfection reagent alone (control). Cells were then incubated for 24h in phenol red-free/serum-free media and then in growing media for further 24h (B) or 48h (C). Data are means ± s.e.m. from n=2-5 (B: control n=2, si scrambled n=5, si β catenin n=3) and n=4 (C) independent experiments. Blot in A shows downregulation of β catenin at the indicated times post transfection.

D-F sh scrambled MCF7 were transfected with siRNA specifically targeting STAT3, non targeting siRNA (si scrambled) or transfection reagent alone (control). Cells were then incubated for 24h in phenol red-free/serum-free media and then in growing media for further 24h (E) or 48h (F). Data are means ± s.e.m. from n=4 (E, except si scrambled, n=3) and n=4 (F) independent experiments. Blot in D shows downregulation of STAT3 48h after transfection (as in E).

Figure S5

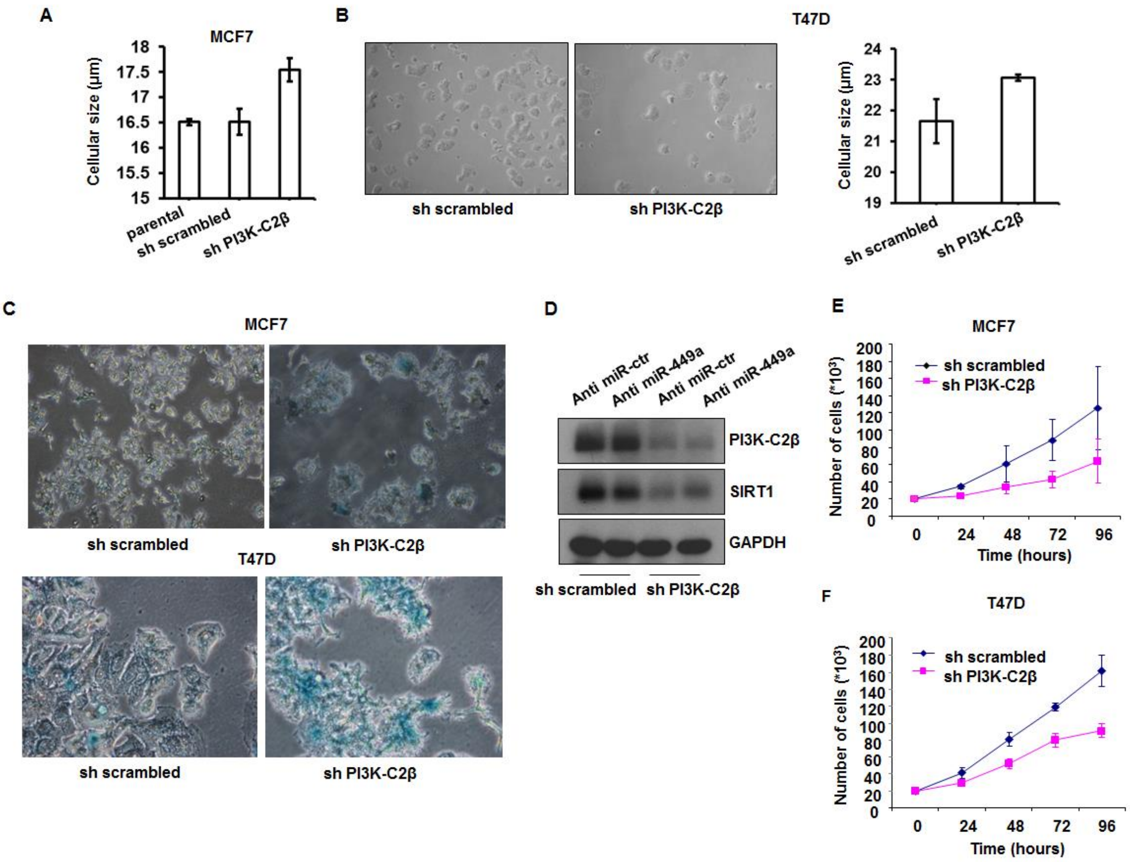

Figure S5: PI3K-C2β regulates cellular senescence via miR-449a regulation.

A Cellular size of the indicated MCF7 cell lines was assessed by FACS analysis.

B Representative images and cellular size of the indicated T47D cells.

C Senescence–associated β-galactosidase assay was performed in sh scrambled and sh PI3K-C2β MCF7 and T47D cell lines. Blue staining indicates accumulation of the senescence marker.

D SIRT1 protein levels were assessed in the same lysates from MCF7 transfected with the indicated anti miRs and presented in Fig 3G. GAPDH was used as loading control.

E Results from cell counting assay in MCF7 and T47D cell lines. Data are means ± s.e.m. of n=3 independent experiments.

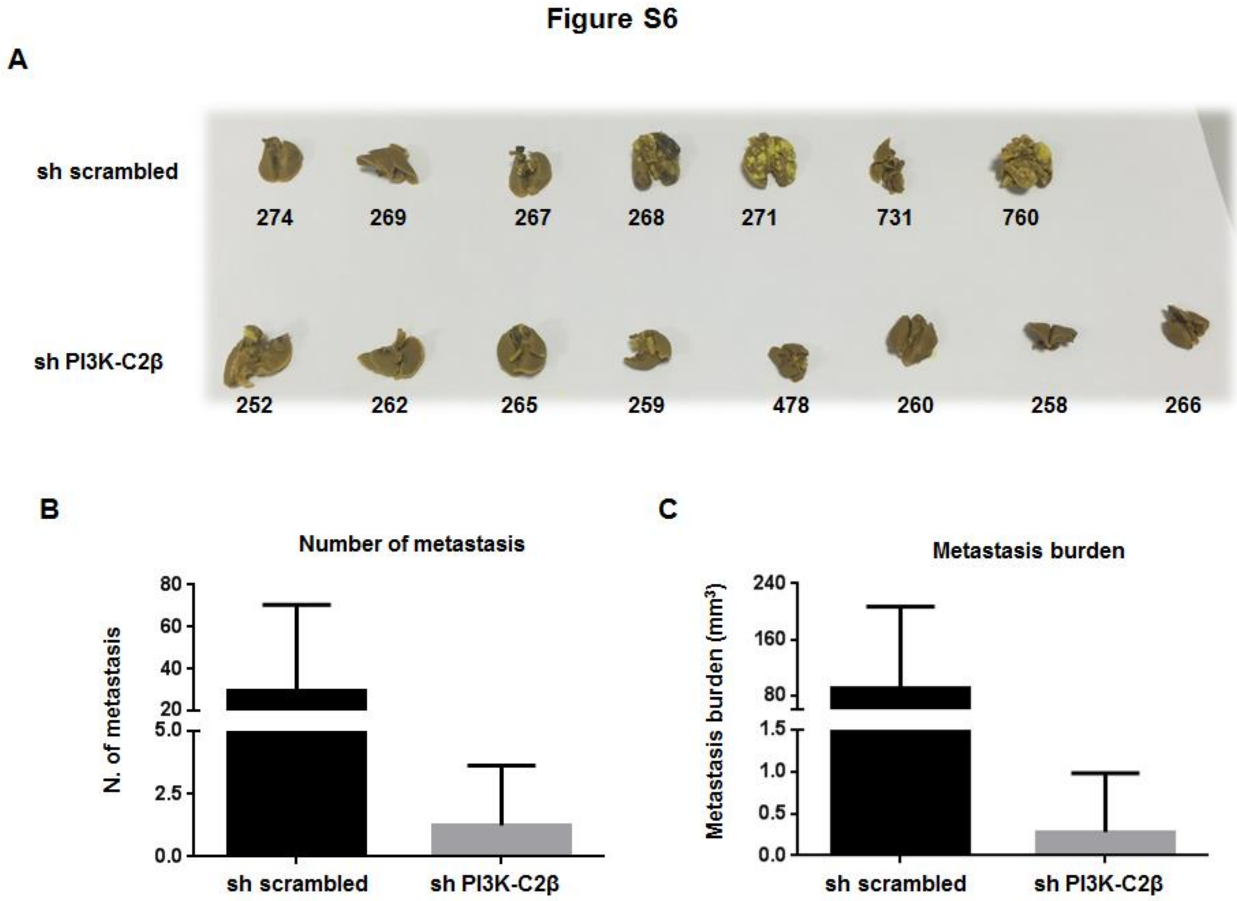

Figure S6: PI3K-C2β regulates breast cancer metastasis formation

A 5 x 10^5 sh scrambled or sh PI3K-C2β MDA-MB-231 cells were injected i.v. and mice were sacrificed the day after the first mouse from group injected with control cells died. Images from excised lungs fixed in Bouin's solution.

B,C Superficial metastatic nodules were counted and measured using a dissecting microscope. The bar represents the mean ±SD of metastasis number (B) and burden (C). Metastasis volume and burden were evaluated as described in the Methods.

Figure S7

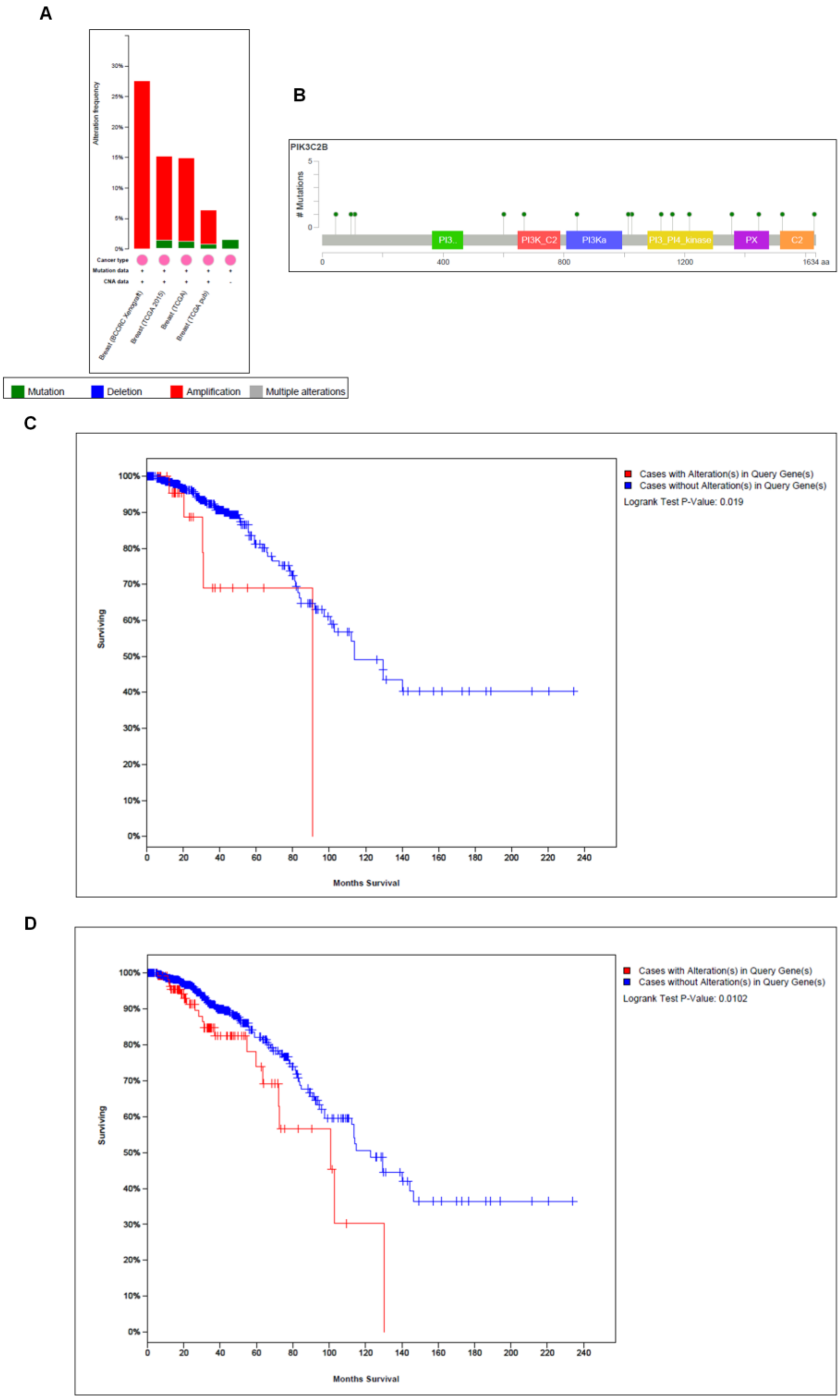

Figure S7: Alteration of *PIK3C2B* in breast cancer

A Frequencies of *PIK3C2B* alterations in 7 breast cancer datasets available on cBioPortal.

B Point mutation sites on the *PIK3C2B* gene.

C Kaplan-Meier overall survival curves for invasive breast cancer cases with (red) or without (blue) *PIK3C2B* amplification [Breast Invasive Carcinoma (TGCA, Nature 2012)]. Number of deceased with *PIK3C2B* alterations: 5 over 31 (16.1%), median months survival 90,75. Number of deceased without *PIK3C2B* alterations: 55 over 451 (12.1%), median months survival 113.74.

D Kaplan-Meier overall survival curves for invasive breast cancer cases with (red) or without (blue) *PIK3C2B* amplification [Breast Invasive Carcinoma (TGCA, Cell 2015)]. Number of deceased with *PIK3C2B* alterations: 21 over 146 (14.4%), median months survival 100.62. Number of deceased without *PIK3C2B* alterations: 90 over 806 (11.1%), median months survival 122.8.

Table S1: Expression of miR-449a in two independent datasets of primary human breast cancers

Array express dataset ID	E-GEOD-19783	E-GEOD-19783	E-GEOD-12848	E-GEOD-12848
miR	hsa-miR-449a	hsa-miR-449a	hsa-miR-449a	hsa-miR-449a
Agilent probe (if available)	A_25_P00010220	A_25_P00010221		
**Basal vs LUM A**	T-test p-value<br>logFC	0.000027<br>-0.900	0.000153<br>-0.638	0.001<br>-7.50
**Basal vs normal-like**	T-test p-value<br>logFC	0.005<br>-0.400	0.005<br>-0.277	0.1038<br>-0.5172
**p53 mut vs p53 wt**	T-test p-value<br>logFC	0.001<br>-0.502	0.002<br>-0.375	0.000054<br>-4.774
**grade 3 vs grade 1+2**	T-test p-value<br>logFC			0.001<br>-5.088
**T3+T4 vs T1**	T-test p-value<br>logFC			0.305<br>-5.408

Table S2: Number of metastasis in the indicated mice injected with sh scrambled and sh PI3K-C2β MDA-MB-231 cells

NUMBER OF METASTASIS

	Diameter (x)					
	**#**	**x≤1 mm**	**1 mm<x≤2 mm**	**2 mm<x≤3 mm**	**x>3mm**	**TOTAL**
**sh scrambled**	**274**	0	0	0	0	0
	**269**	1	1	0	0	2
	**267**	0	0	0	0	0
	**268**	57	20	5	3	85
	**271**	37	35	12	5	89
	**731**	0	0	0	0	0
	**760**	12	9	2	9	32
					**median**	**2**
					**mean**	**29.71**
					**st dev**	**40.81**

	Diameter (x)					
	**#**	**x≤1 mm**	**1 mm<x≤2 mm**	**2 mm<x≤3 mm**	**x>3mm**	**TOTAL**
**sh PI3K-C2β**	**252**	4	0	0	0	4
	**262**	5	1	0	0	6
	**265**	0	0	0	0	0
	**259**	0	0	0	0	0
	**478**	0	0	0	0	0
	**260**	0	0	0	0	0
	**258**	0	0	0	0	0
	**266**	0	0	0	0	0
					**median**	**0**
					**mean**	**1.25**
					**st dev**	**2.38**

Table S3: Metastasis tumor burden in the indicated mice injected with sh scrambled and sh PI3K-C2β MDA-MB-231 cells

METASTASIS TUMOR BURDEN

	Diameter (x)					
	**#**	**x≤1 mm**	**1 mm<x≤2 mm**	**2 mm<x≤3 mm**	**x>3mm**	**TOTAL**
	**274**	0	0	0	0	0
	**269**	0.06	1.69	0	0	1.75
**sh**	**267**	0	0	0	0	0
**scrambled**	**268**	3.56	33.75	39.06	64.31	140.69
	**271**	2.31	59.06	93.75	107.19	262.31
	**731**	0	0	0	0	0
	**760**	0.75	15.19	15.63	192.94	224.50
					**median**	**1.75**
					**mean**	**89.89**
					**st dev**	**117.22**

	Diameter (x)					
	**#**	**x≤1 mm**	**1 mm<x≤2 mm**	**2 mm<x≤3 mm**	**x>3mm**	**TOTAL**
	**252**	0.25	0	0	0	0.25
	**262**	0.3125	1.6875	0	0	2
	**265**	0	0	0	0	0
**sh**	**259**	0	0	0	0	0
**PI3K-C2β**	**478**	0	0	0	0	0
	**260**	0	0	0	0	0
	**258**	0	0	0	0	0
	**266**	0	0	0	0	0
					**median**	**0**
					**mean**	**0.28**
					**st dev**	**0.70**
